# Integrative taxonomy reveals cryptic diversity within the *Euphorbia nicaeensis* alliance (Euphorbiaceae) in the central Balkan Peninsula

**DOI:** 10.3389/fpls.2025.1558466

**Published:** 2025-04-14

**Authors:** Angela Sharovikj Ivanova, Peter Schönswetter, Mitko Kostadinovski, Michael H. J. Barfuss, Renata Ćušterevska, Božo Frajman

**Affiliations:** ^1^ Institute of Biology, Faculty of Natural Sciences and Mathematics, Ss. Cyril and Methodius University Skopje, Skopje, North Macedonia; ^2^ Department of Botany, University of Innsbruck, Innsbruck, Austria; ^3^ Department of Botany and Biodiversity Research, University of Vienna, Vienna, Austria

**Keywords:** biodiversity, Balkan Peninsula, extrazonal steppes, new species, phylogeny, polyploidy, Pontic steppes, taxonomy

## Abstract

The Eurasian steppes are the second-largest continuous biome on Earth. *Euphorbia glareosa*, a member of the *Euphorbia nicaeensis* alliance, is a widespread species in the macroclimatically defined zonal Pontic steppes and westerly and southerly adjacent extrazonal steppe outposts determined by local climatic, topographic, and edaphic conditions. In the extrazonal steppes, in particular within the Anatolian, Danubian, Thracian, and Pannonian grasslands, it is more or less continuously distributed, but with several disjunct occurrences in the central Balkan Peninsula, which is renowned for its high biodiversity. Several (infra)specific taxa have been recognised within *E. glareosa* s.l., but relationships among them remain elusive. We applied an integrative approach ranging from cytogenetics (relative genome size and ploidy estimation, chromosome counting) and morphometrics to phylogenetics (internal ribosomal transcribed spacer sequencing and amplified length polymorphism fingerprinting), with geographic focus on the central and eastern Balkan Peninsula. We inferred multiple polyploidisations within the group and complex phylogenetic patterns. We uncovered cryptic lineages in the central Balkan Peninsula, where the description of two new species, diploid *E. balcanica*, and tetraploid *E. skopjensis* was necessary. In addition, we revealed high diversity, partly related to polyploidisations, among the populations from the eastern Balkan and Pontic steppes, likely pertaining to different species. Finally, the main phylogeographic split within *E. glareosa* is between (1) Pannonian, central and eastern Balkan populations, and (2) the easternmost Balkan, Pontic, and Anatolian populations. Our results thus highlight the outstanding conservation value of the extrazonal European steppes that are not just an outpost of zonal Eurasian steppes. We also point to the remarkable biodiversity of the central and eastern Balkan Peninsula and the need for further in-depth studies of this biodiversity hotspot.

## 1 Introduction

The Eurasian steppes are the second-largest continuous biome on Earth spanning from Central and Eastern Europe (Pannonian and Pontic areas) to Central and northeastern Asia (Lal, 2004; Wesche et al., 2016; Kirschner et al., 2020). They represent various types of temperate grasslands (Coupland, 1993), which are shaped by strongly seasonal climates and cold winters (Peart, 2008). They are similar in several characteristics to the grasslands of Mediterranean mountain ranges, and several plant genera and species extend across both biomes (Hamasha et al., 2012; Stojilkovič et al., 2022). The Balkan Peninsula represents a link between extensive steppe areas in the East, and the Mediterranean Basin in the South and West, and thus a crossroad of floras from both areas (Tomović et al., 2014). With the exception of the northeasternmost Balkan Peninsula (Dobrudja), which is considered a part of the macroclimatically defined Eurasian zonal steppes, extrazonal steppe outposts determined by local climatic, topographic, and edaphic conditions are disjunctly distributed in the eastern and southern parts of the Balkans. Within the extrazonal steppes of the Balkan Peninsula, the Danubian and Thracian lowlands are covered with wide-ranging steppe vegetation, whereas smaller grassland areas embedded in a matrix of forest vegetation are characteristic of westerly adjacent areas (Wesche et al., 2016; Kirschner et al., 2020). The eastern and southern Balkans are also the region that, compared to the western and central parts, remained largely neglected in phylogenetic studies (Španiel and Rešetnik, 2022); the few existing studies dedicated to or including plants from these areas point to high intra- as well as interspecific diversity, e.g., in *Astragalus onobrychis* L. (Fabaceae; Záveská et al., 2019), *Aurinia saxatilis* (L.) Desv. (Brassicaceae; Rešetnik et al., 2022), *Cerastium decalvans* Schloss. & Vuk. (Caryophyllaceae; Niketić et al., 2022), *Cyanus tuberosus* (Vis.) Soják group (Asteraceae; Skokanová et al., 2019), *Sesleria rigida* Heuff. complex (Poaceae; Kuzmanović et al., 2013), and *Silene saxifraga* alliance (Caryophyllaceae; Đurović et al., 2017). In addition, description of several new species from the central parts of the Balkan Peninsula in the last decades (reviewed in Frajman et al., 2014) points to a lack of contemporary biodiversity studies in this area.

One of the widespread steppe species, more or less continuously distributed in the Pontic and Pannonian, as well as the Danubian and Thracian grasslands, with several disjunct occurrences in the central Balkan Peninsula (Bosnia and Herzegovina, North Macedonia, Serbia), as well as in Anatolia and the Caucasus, is *Euphorbia glareosa* Pall. ex M. Bieb. This species was considered conspecific with the Mediterranean *E. nicaeensis* All. by Radcliffe-Smith and Tutin (1968), but Stojilkovič et al. (2022) and Boschin et al. (2024) have shown that these taxa are clearly divergent, and three species should be recognised in the Mediterranean Basin as follows: the western Mediterranean *E. nicaeensis*, the central Mediterranean (Apennine Peninsula and north-western Balkan Peninsula) *E. japygica* Ten., and the western Balkan endemic *E. hercegovina* Beck. They all, along with *E. glareosa* and some species distributed in the Irano-Turanian region, belong to the *E. nicaeensis* alliance (Stojilkovič et al., 2022).

On the other hand, *E. glareosa* was shown to constitute an assemblage of populations with different genome sizes and putatively different ploidies (Stojilkovič et al., 2022). This, along with pronounced morphological variability that led to the recognition of different taxa in the past (e.g., Prokhanov, 1949; Kuzmanov, 1979; Greuter et al., 1986; Geltman, 2009), suggests that *E. glareosa* is a species complex including multiple taxa rather than a single species. Stojilkovič et al. (2022) treated this assemblage as *E. glareosa* s.l., which includes seven taxa with unclear taxonomic status and relationships as follows: *E. cadrilateri* Prodan, *E. dobrogensis* Prodan, *E. glareosa*, *E. goldei* Prokh., *E. pannonica* Host., *E. stepposa* Zoz, and *E. volgensis* Krysth. These taxa were in the past partly treated as subspecies (e.g., Kuzmanov, 1979; Radcliffe-Smith, 1982; Greuter et al., 1986; Govaerts et al., 2000) or species (e.g., Prodan, 1936; Prokhanov, 1949; Geltman, 2009, 2020). We apply the name *E. glareosa* s.l. for all these taxa hereafter.

Although most populations of *E. glareosa* s.l. formed a monophyletic group in the phylogenetic trees based on the restriction site-associated DNA sequencing (RADseq) data, one population from the Skopje basin in North Macedonia was phylogenetically divergent. It appeared more closely related to the Mediterranean species of the *E. nicaeensis* alliance and Irano-Turanian *E. macroclada* Boiss. (Stojilkovič et al., 2022). This population had the highest relative genome size (RGS) of all studied populations, indicating its polyploid origin, which was assumed to explain its divergent phylogenetic position (Stojilkovič et al., 2022). In addition, within the *E. glareosa* clade, two populations from (1) Dobrudja and (2) Armenia, with higher genome size than most populations of *E. glareosa*, were phylogenetically divergent and sister to the other populations with weak support (Stojilkovič et al., 2022). Finally, isolated populations from northern Albania that were treated as *E. nicaeensis* (incl. *E. glareosa* s.l.) by Qosja et al. (1992) and Barina (2017) were not studied by Stojilkovič et al. (2022), who suggested that they might be more closely related to *E. japygica* than to *E. glareosa* s.l.

Given the high phylogenetic, RGS, and morphological variability within *E. glareosa* s.l., our aim is to disentangle the relationships among the populations currently classified as *E. glareosa* s.l., with geographic focus on the central and eastern Balkan Peninsula, including populations from north Albania and based on an extensive sampling in Bulgaria, North Macedonia, and Serbia. We sampled multiple populations that could pertain to different taxa of *E. glareosa* s.l. across this area and used an integrative approach to infer the relationships among them and to clarify their taxonomic status. More specifically, we (1) estimated the ploidy of all investigated populations via relative genome size (RGS) estimation with flow cytometry and chromosome counting of selected populations. (2) Using nuclear ITS sequences and amplified fragment length polymorphism (AFLP) fingerprinting, we inferred the origin of, and the phylogeographic differentiation within, the study species. Finally, (3) using multivariate morphometrics, we explored the morphological differentiation, and (4) based on all data, we propose a revised taxonomic treatment. Since RGS and AFLP analyses revealed clear divergence of populations from the central Balkan Peninsula (Albania, Kosovo, North Macedonia) that were also morphologically divergent from *E. glareosa* s.l. (see Results), we describe them below as two new taxa, diploid *E. balcanica* and tetraploid *E. skopjensis*. For simplicity, we apply these names hereafter.

## 2 Materials and methods

### 2.1 Plant material

**Figure 1:**
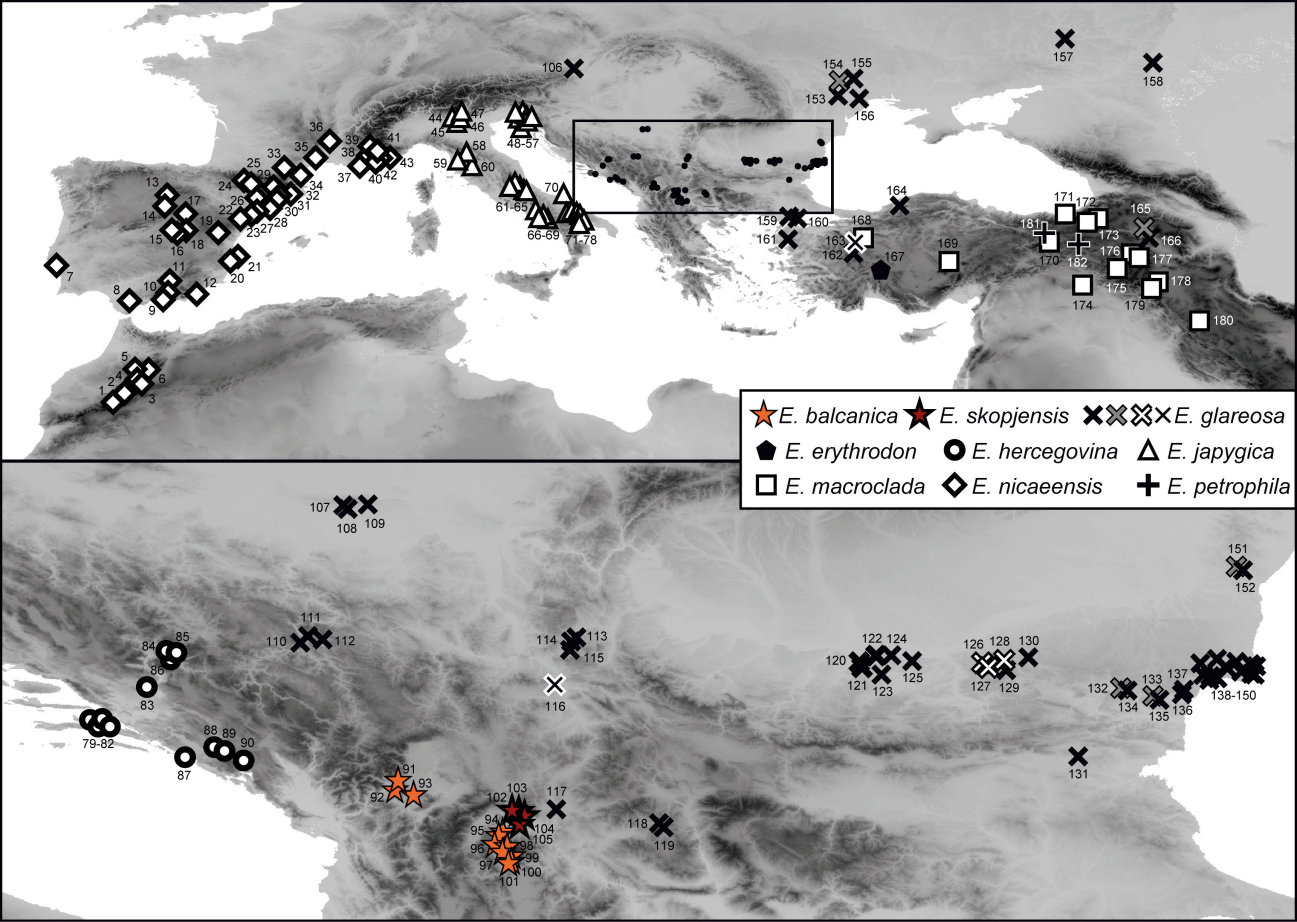
Populations of *Euphorbia balcanica* (populations 91–101), *E. skopjensis* (102–105), and other species from the *E. nicaeensis* alliance used in this study. The area within the frame in the upper panel is enlarged in the lower panel. Population numbers correspond to **Supplementary Table S1**. Different symbols of *E. glareosa* s.l. represent populations with different relative genome size/ploidy.

We collected plant material from *E. balcanica*, *E. skopjensis*, *E. glareosa* s.l., and outgroup taxa (leaves dried in silica gel, herbarium vouchers, and seeds) for molecular, morphometric, and karyological (RGS and chromosome number estimation) analyses in the field between 2006 and 2024. We studied a total of 11 populations of *E. balcanica*, 4 of *E. skopjensis*, and 62 of *E. glareosa* s.l. In total, 5 populations of *E. balcanica* and 3 of *E. skopjensis* were included in AFLP analyses, 4/2 in ITS analyses, all in RGS analyses, and 10/4 in morphometric analyses. For population 95 of *E. balcanica* and 105 of *E. skopjensis*, we counted the chromosomes. For *E. glareosa* s.l., 32 populations were included in ITS (16 from Stojilkovič et al., 2022), 51 (18) in RGS analyses, 37 in AFLP, and 83 (16) in morphometric analyses, respectively, and for population 118, we counted the chromosomes (**Figure 1**; **Supplementary Table S1**).

### 2.2 Relative genome size and ploidy-level estimation

We measured the RGS using a CyFlow space flow cytometer (Partec, GmbH, Münster, Germany) following the procedure of Suda and Trávníček (2006). Nuclei from silica gel-dried leaf material of our samples (**Supplementary Table S1**) and fresh leaves of the reference standard *Bellis perennis* L. (2C = 3.38 pg; Schönswetter et al., 2007) were stained using 4′,6-diamidino-2-phenylindole (DAPI). In cases where the peaks of the reference standard and the sample overlapped, *Pisum sativum* L. (2C = 8.84 pg; Greilhuber and Ebert, 1994) was used as secondary standard. In such cases, we first measured the RGS of both standards together, and then that of the secondary standard and the sample. We then recalculated the RGS of the sample and displayed it in relation to the primary standard. We recorded the relative fluorescence of 3,000 nuclei for three to five individuals per population and used FloMax software (Partec) to evaluate histograms and calculate coefficients of variation. The RGS was calculated as the ratio between the values of the mean relative fluorescence of the sample and the standard. We used the visualisation package “ggplot2” in RStudio 1.2.5019 (RStudio Team, 2022, version R-3.6.1) to produce box plots of RGS. In addition to *E. balcanica*, *E. skopjensis*, and *E. glareosa* s.l., we included the RGS data of closely related *E. erythrodon* Boiss. & Heldr., *E. hercegovina*, *E. japygica*, *E. macroclada*, *E. nicaeensis*, and *E. petrophila* C. A. Mey. from Stojilkovič et al. (2022) and Boschin et al. (2024).

### 2.3 Chromosome number estimation

Seeds collected in the field were incubated at 4°C for 2 weeks. After removal of the caruncle, the seeds were sterilised with a mixture of bleach and water 1:3 for 10 min to inhibit fungal growth and then incubated on sterile paper in Petri dishes. Root tips of germinated seeds were pretreated with 0.002 M colchicine for 2 h at room temperature and then for 2 h at 4°C, fixed in Carnoy’s solution (3:1 mixture of ethanol and acetic acid) for 24 h at 4°C and then stored in ethanol at −21°C.

Hydrolysis was performed in 5 N HCl for 60 min at room temperature. The tips were stained with Feulgen’s reagent, kept in the dark for 2 h at room temperature and then rinsed with water. Slides were prepared by squashing the stained meristem in a drop of 45% acetic acid under the coverslip. Slides were then snap frozen, dehydrated in 96% ethanol for 5 min and air dried. Chromosomes were counted using a Nikon Eclipse 80i microscope; images were taken using a Canon 600D camera and processed using Canon EOS Utility software.

### 2.4 DNA extraction, ITS sequencing, and analysis of sequence data

Total genomic DNA extraction and ITS sequencing were performed as described by Frajman and Schönswetter (2011). Sequencing was performed at Eurofins Genomics (Ebersberg, Germany). Contigs were assembled, edited, and sequences aligned using Geneious Pro 5.5.9 (Kearse et al., 2012). Base polymorphisms were coded using NC-IUPAC ambiguity codes. Twenty-one ITS sequences were produced in this study. In addition, 60 ITS sequences of the outgroup taxa and 16 of *E. glareosa* s.l., as well as 1 of *E. skopjensis* were from Stojilkovič et al. (2022), and 8 of *E. japygica* were from Boschin et al. (2024). Genbank numbers are in **Supplementary Table S1**. Maximum parsimony (MP) and MP bootstrap (MPB) analyses were performed using PAUP v4.0b10 (Swofford, 2002) as described by Frajman et al. (2019). Bayesian analyses were performed using MrBayes 3.2.1 (Ronquist et al., 2012) using the HKY+Γ substitution model and settings such as in Frajman et al. (2019). We also created a NeighbourNet with ITS sequences of *E. balcanica*, *E. skopjensis*, and the most closely related taxa using SplitsTree4 12.3 (Huson and Bryant, 2006).

### 2.5 AFLP analyses

The AFLP procedure followed that of Vos et al. (1995) with modifications described by Cresti et al. (2019). In addition to 5 populations of *E. balcanica*, 3 of *E. skopjensis*, and 34 of *E. glareosa* s.l., we included 1 population of *E. erytrodon*, 2 of *E. hercegovina*, 2 of *E. japygica*, 2 of *E. macroclada*, 2 of *E. nicaeensis*, and 1 of *E. petrophila* as outgroups, based on the study of Stojilkovič et al. (2022).

The three primers for selective PCR (fluorescent dye in brackets) were EcoRI (FAM)-ATG/MseI-CTT, EcoRI (VIC)-ACG/MseI-CAA, and EcoRI (NED)-ACC/MseI-CAG. Two microliters of the elution product was mixed with 10 µl of formamide and 0.1 µl of GeneScan ROX (ThermoFisher Scientific) and run on a 3130*xl* Genetic Analyzer (Applied Biosystems). A blank (DNA replaced by water) was included to test for systematic contamination, and 16 samples were used as replicates between the two PCR batches to evaluate the reproducibility of the method.

Electropherograms were analysed with Peak Scanner 1.0 (Applied Biosystems) using default peak detection parameters. Automated binning and scoring of AFLP fragments was performed using RawGeno 2.0-1 (Arrigo et al., 2009) for RStudio 2022.12.0 + 353 (RStudio Team, 2022) with the following settings: scoring range 75–400 bp, minimum intensity 100 RFUs, minimum bin width 1 bp, and maximum bin width 1.5 bp. Fragments with reproducibility <80% based on sample-replicate comparisons were eliminated. The error rate calculated in RawGeno based on 16 sample–replicate comparisons was 3.5%. Finally, after exclusion of nine individuals that failed to produce reliable fingerprints, a matrix of 167 individuals was generated. In addition, we produced a dataset of 114 individuals, including only *E. glareosa* s.l., using the same settings as above; in this case, the error rate was 2.9%.

A neighbour-joining (NJ) analysis based on Nei–Li genetic distances (Nei and Li, 1979) was performed and bootstrapped (2,000 pseudo-replicates) with TREECON 1.3b (Van de Peer and de Wachter, 1997) for both AFLP datasets. In the first dataset, we used *E. nicaeensis* for rooting, and, in the second dataset, the population 165 of *E. glareosa* that was the most early divergent in the NJ tree of the first dataset (see results). In addition, for the second dataset, including all populations of *E. glareosa* s.l., we used SplitsTree4 12.3 (Huson and Bryant, 2006) to create a NeighbourNet based on uncorrected P distances, as well as non-hierarchical K-means clustering (Hartigan and Wong, 1979) with a script by Arrigo et al. (2010) in RStudio 2022.12.0 + 353 (RStudio Team, 2022). In the last analysis 50,000 independent runs were performed (i.e., starting from random points) for each assumed value of K clusters ranging from 2 to 10. To select the best number of groups, the strategy proposed by Evanno et al. (2005) was used, and the proportions of individuals assigned to the K-means groups were plotted for populations on a map in ArcMAP 10.8.2 (ESRI, 2021).

### 2.6 Morphometric analyses

We performed morphometric analyses on 28 individuals from 10 populations of *E. balcanica* and 24 individuals from 4 populations of *E. skopjensis* as well as 83 individuals from 47 populations of *E. glareosa* s.l. In total, we measured 33 characters and calculated 15 ratios (**Supplementary Table S2**). Stem and leaf characters were measured manually. All other characters (cyathium, fruit, and seed characters) were measured on images taken with an Olympus SZX9 stereomicroscope (Olympus Gmbh, Hamburg, Germany) using the Olympus image analysis software analySIS pro. Our measurements were supplemented by morphometric data of 15 individuals from 15 populations of *E. glareosa* s.l., 20 individuals from 13 populations of *E. hercegovina*, and 1 individual of *E. skopjensis* from Stojilkovič et al. (2022), as well as 45 individuals from 33 populations of *E. japygica* from Boschin et al. (2024). A total of 14 individuals of *E. balcanica*, 3 of *E. glareosa* s.l., and 15 of *E. hercegovina* did not have fully developed fruits and seeds.

Statistical analyses were performed using SPSS 24.0 (IBM Corp., Armonk, NY), separately for (1) vegetative parts of the plants and cyathium characters, and (2) fruit and seed characters for (A) all abovementioned taxa as well as (B) for *E. balcanica* and *E. skopjensis*. Correlations between metric characters were tested using Pearson and Spearman correlation coefficients. In the vegetative dataset of all taxa (1A), the correlation coefficients exceeded 0.82 for the following character pairs: length of a middle stem leaf–distance from base to widest part of stem leaf, length of a ray leaf–distance from the base to the widest part of a ray leaf, depth of gland emargination–ratio depth of gland emargination/length of cyathial gland, and length of the longest terminal ray–length of the longest fertile axillary ray. In the vegetative dataset of *E. balcanica* and *E. skopjensis* (1B), the correlation coefficients exceeded 0.82 for the following character pairs: length of a middle stem leaf–distance from base to widest part of stem leaf, length of cyathial gland–ratio of length/width of a cyathial gland, length of the longest terminal ray–width of a raylet leaf, length of a raylet leaf–width of a raylet leaf, depth of gland emargination–ratio depth of gland emargination/length of cyathial gland, length of the longest terminal ray–length of a raylet leaf, length of a ray leaf–length of a raylet leaf, length of a ray leaf–width of a raylet leaf. The latter character of each character pair in all cases was excluded from further analyses. In the fruit and seed character sets, the correlation coefficients were lower than 0.82; therefore, all characters were kept for further analyses.

After standardisation to 0 mean and 1-U variance, principal component analysis (PCA) was performed. This was followed by discriminant analysis (DA). We also produced BoxPlots for the most differentiating characters. Finally, based on the morphometric data, we created taxon descriptions and an identification key. The metric values shown correspond to the 10th and 90th percentiles supplemented by extreme values in parentheses.

## 3 Results

### 3.1 Relative genome size and chromosome number

**Figure 2:**
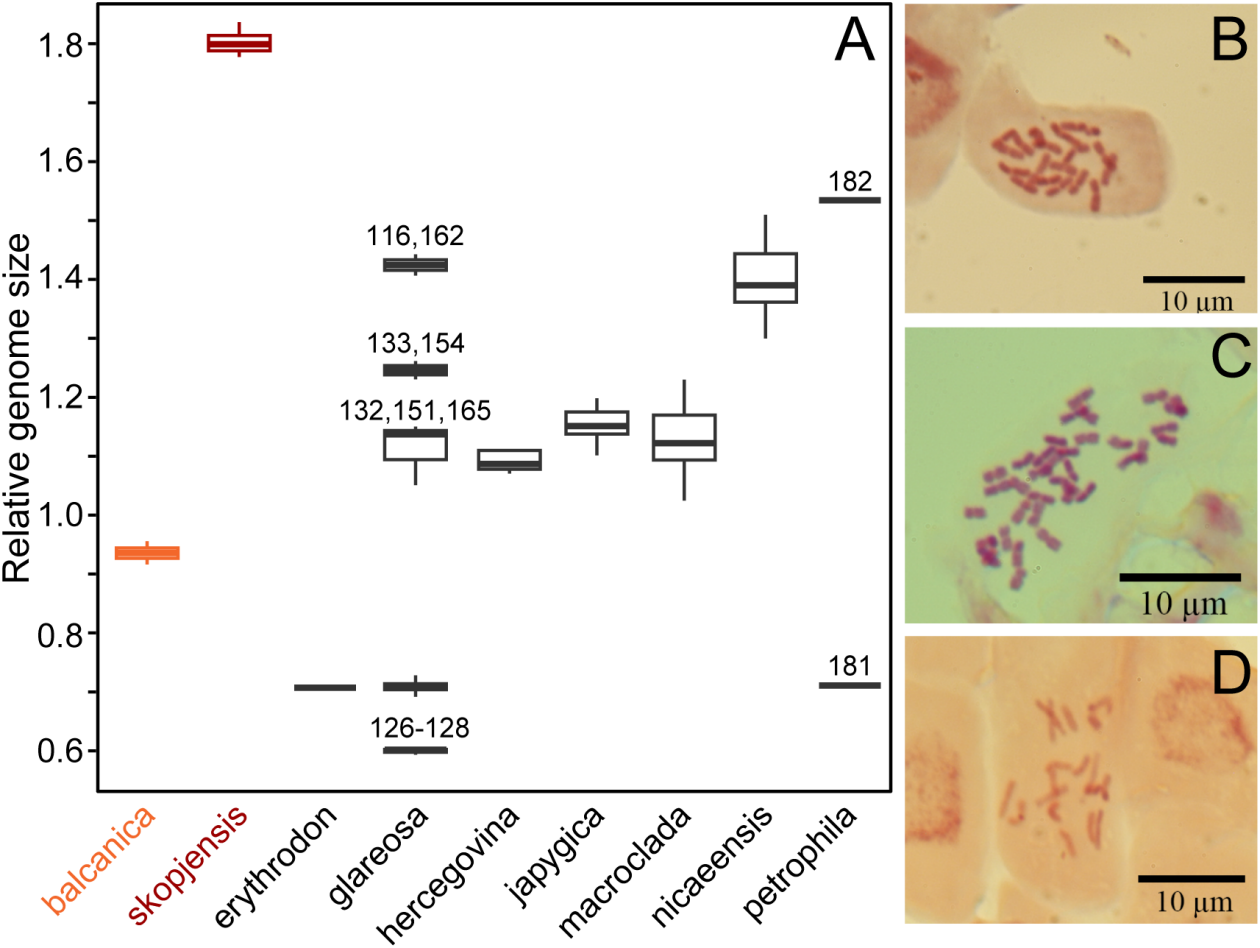
**(A)** Relative genome size (RGS) variation in *Euphorbia balcanica* and *E. skopjensis*, as well as other species from the *E. nicaeensis* alliance. Metaphase chromosome plates of **(B)** *E. balcanica* from population 95 with 2*n* = 18, **(C)** *E. skopjensis* from population 105 with 2*n* = 36, and **(D)** *E. glareosa* from population 118 with 2*n* = 18.

The RGS of *E. balcanica* was between 0.895 and 0.953 and that of *E. skopjensis* between 1.771 and 1.830 (**Supplementary Table S1**; **Figure 2A**). The first range corresponded to diploids with 2*n* = 18 (population 95; **Figure 2B**) and the second to tetraploids with 2*n* = 36 (population 105; **Figure 2C**). Within *E. glareosa* s.l., multiple RGS values were detected. The majority (41) of populations had RGS between 0.683 and 0.731. Among them was also population 118, for which we counted 18 chromosomes (**Figure 2D**). Populations 116 from Serbia and 162 from Turkey had RGS 1.401 and 1.436, respectively, which likely corresponds to tetraploids. In addition, several populations had deviating RGS values: those of populations 126–128 ranged between 0.592 and 0.601, those of populations 132, 151, and 165 between 1.047 and 1.146, and populations 133 and 154 had RGS 1.226 and 1.257, respectively (**Figures 1**, **2**; **Supplementary Table S1**).

### 3.2 Phylogenetic relationships based on internal transcribed spacer sequences

**Figure 3:**
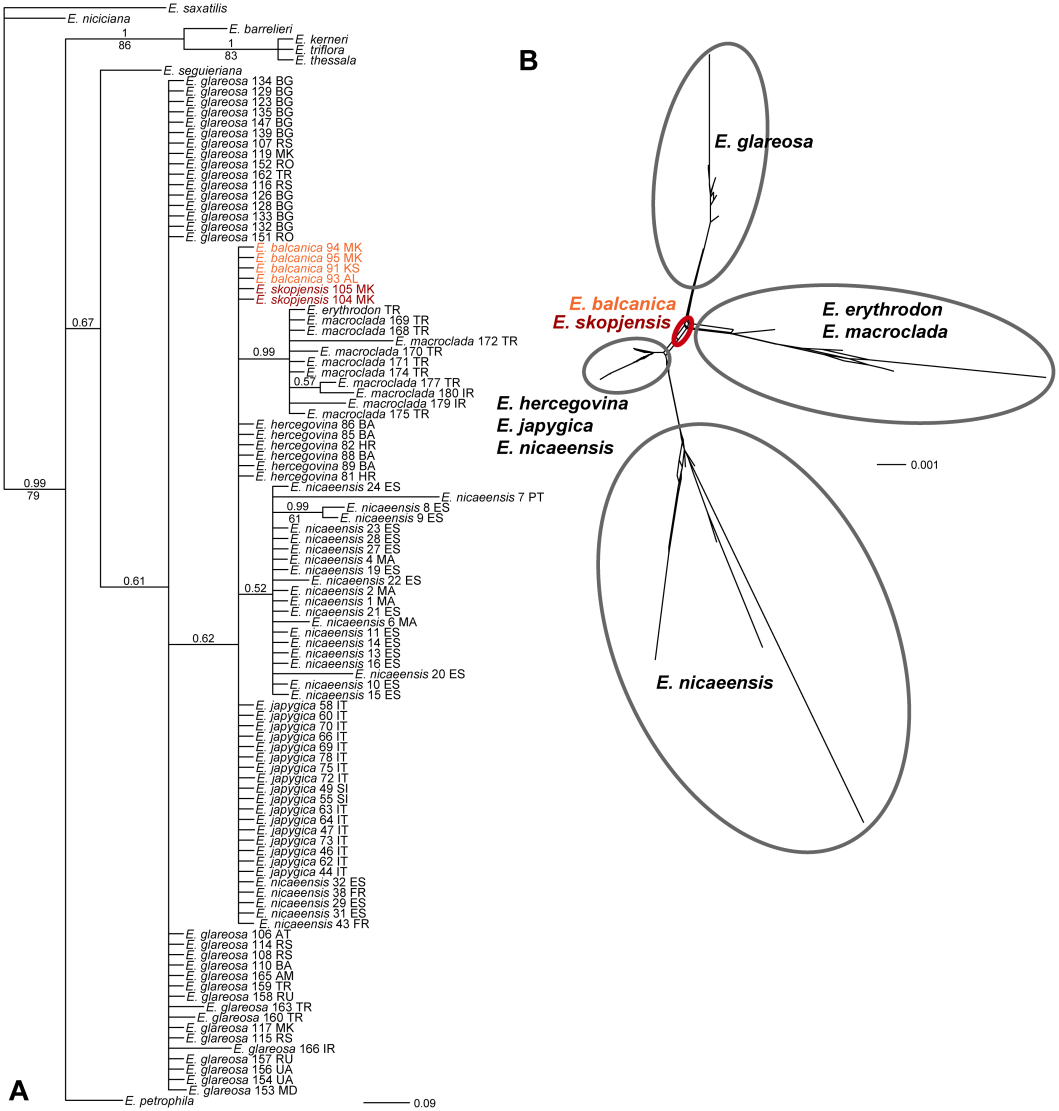
Phylogenetic relationships showing the positions of *Euphorbia balcanica* and *E. skopjensis* within the *E. nicaeensis* alliance inferred by internal transcribed spacer (ITS) sequences. **(A)** Bayesian consensus phylogram. Numbers above branches are posterior probabilities >0.50; those below branches are bootstrap values >50%. Population numbers correspond to those of **Figure 1** and **Supplementary Table S1**. Country codes follow the accession names. **(B)** NeighbourNet.

The relationships in the ITS tree were poorly resolved (**Figure 3A**). Most accessions of *E. glareosa* s.l. were in a basal polytomy in a poorly supported clade (posterior probabilities, PP 0.61), including species of the *E. nicaeensis* alliance (*sensu* Stojilkovič et al., 2022). In this polytomy, there was another poorly supported clade (PP 0.62), including *E. balcanica*, *E. hercegovina*, *E. japygica*, *E. skopjensis*, as well as a clade (PP 0.99) with *E. erythrodon* and *E. macroclada*. In the NeighborNet (**Figure 3B**), *E. balcanica* and *E. skopjensis* had a central position, from which four main splits led to (1) *E. glareosa* s.l., (2) *E. erythrodon* and *E. macroclada*, (3) *E. nicaeensis*, and (4) *E. hercegovina*, *E. japygica*, and *E. nicaeensis*.

### 3.3 Phylogenetic relationships based on amplified fragment length polymorphisms

**Figure 4:**
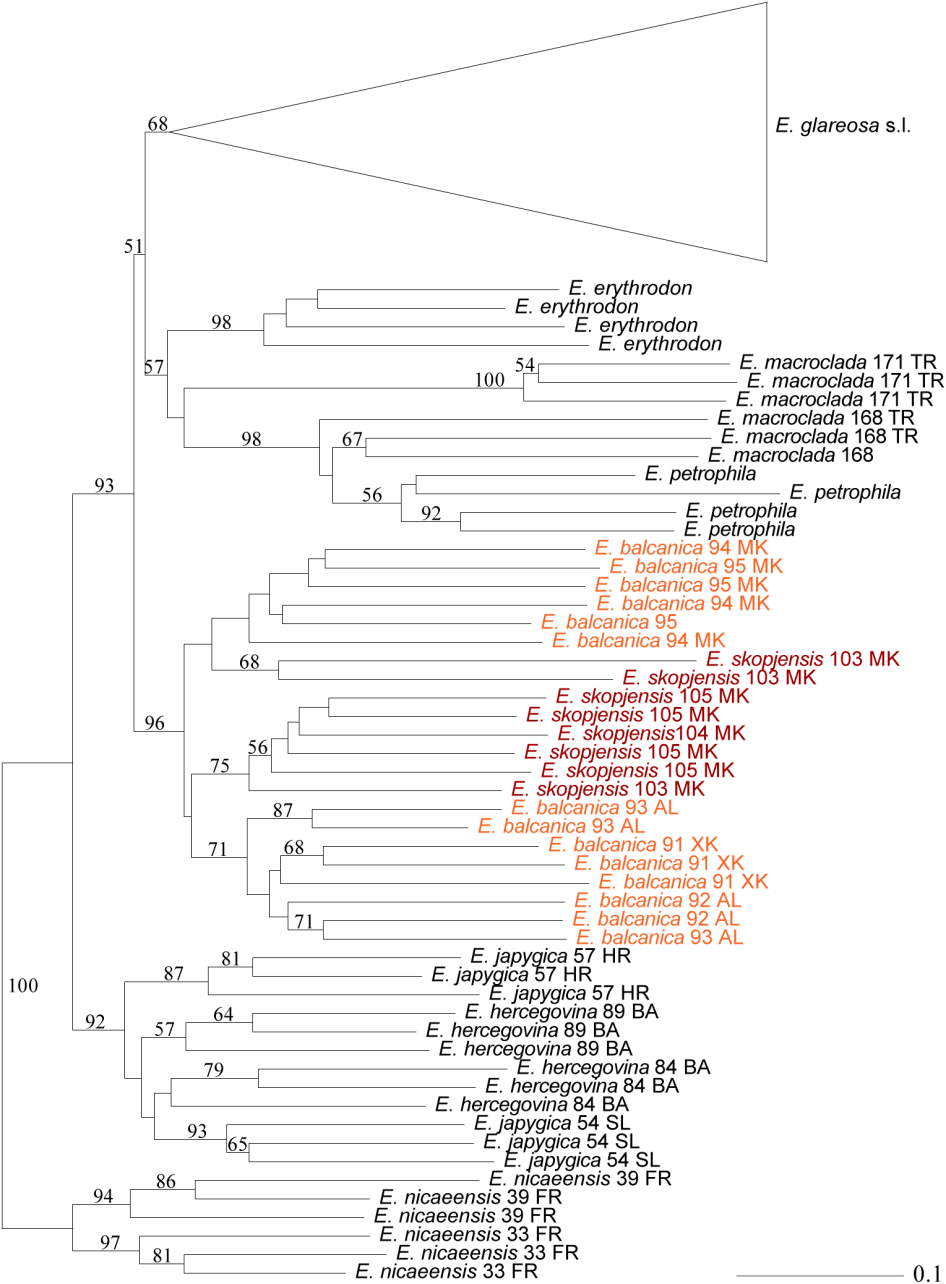
Neighbour Joining tree based on AFLP data showing the phylogenetic position of *Euphorbia balcanica* and *E. skopjensis* within the *E. nicaeensis* alliance. Numbers above branches are bootstrap values >50. Population numbers correspond to those of **Figure 1** and **Supplementary Table S1**. The complete tree with all accessions of *E. glareosa* s.l. is in **Supplementary Figure S1**.

A total of 553 fragments were scored in 167 individuals of the complete dataset and 421 fragments in *E. glareosa* s.l. Of these, 59 and 45 fragments were excluded because they were present or absent in a single individual only. In the neighbour-joining (NJ) tree (**Supplementary Figure S1**; **Figure 4**), *E. hercegovina* and E*. japygica* formed a cluster (bootstrap support, BS 92%) sister to a cluster (BS 93%) containing all other accessions with the exception of *E. nicaeensis* that was used for rooting. In the latter cluster, *E. balcanica* and *E. skopjensis* (BS 96%) were sister to a cluster (51%) with the remaining taxa, corresponding to a cluster (57%) composed of *E. erythrodon*, *E. macroclada*, and *E. petrophila*, and a cluster (BS 68%) containing all accessions of *E. glareosa* s.l. The relationships between *E. balcanica* and *E. skopjensis* were unresolved as two individuals from population 103 of *E. skopjensis* were divergent from all other accessions that formed a cluster with BS 75%. The geographically disjunct populations of *E. balcanica* from Albania and Kosovo (BS 71%) were divergent from those of North Macedonia (BS <50%).

**Figure 5:**
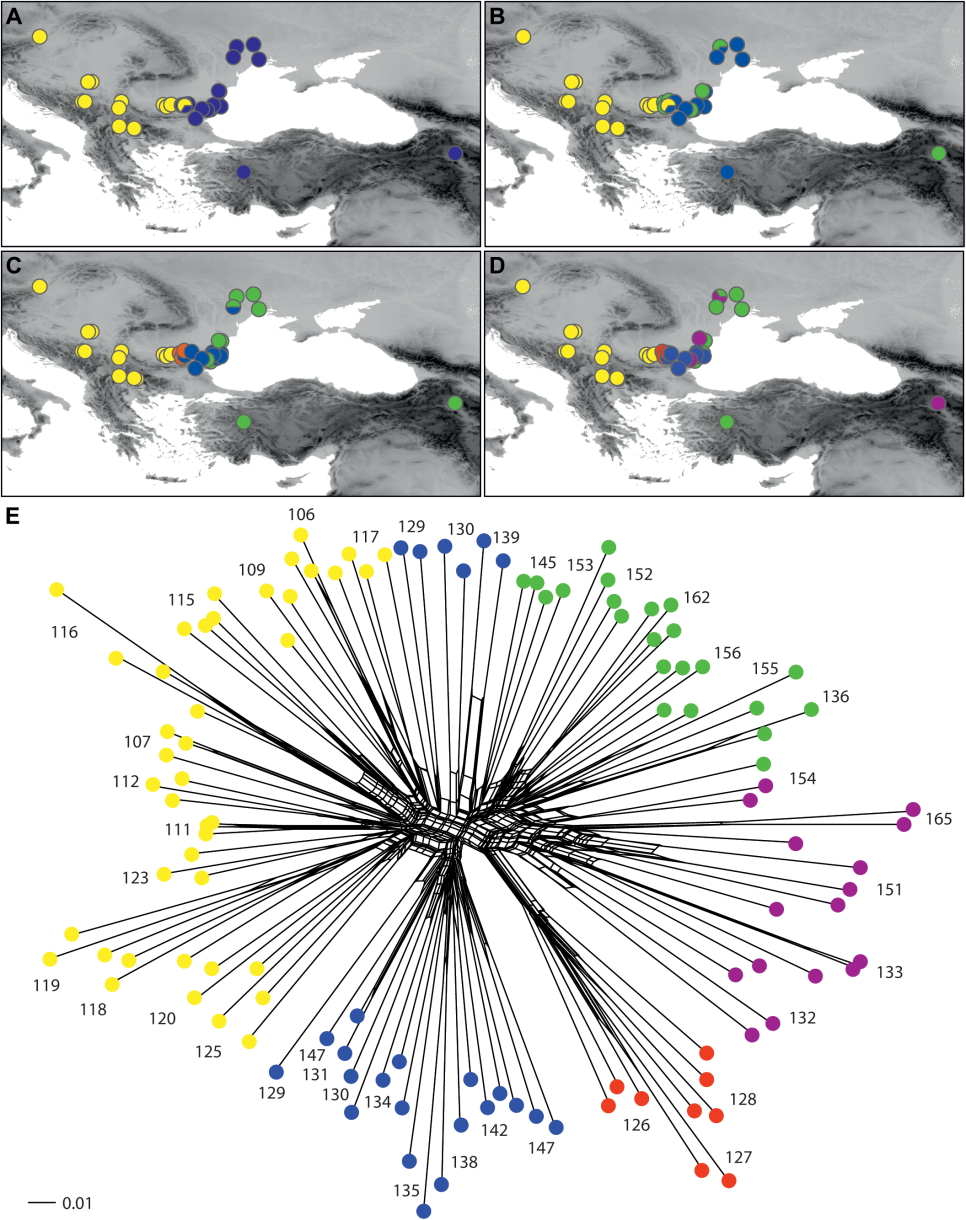
Phylogenetic relationships among populations of *Euphorbia glareosa* s.l. inferred by AFLP fingerprinting. **(A–D)** Geographical distribution of phylogroups inferred by non-hierarchical K-means clustering at K = 2 (optimal division), K = 3, K = 4, and K = 5. **(E)** NeighbourNet based on uncorrected P distances; coloured dots indicate the five groups inferred at K = 5 (as in D). Population numbers correspond to those of **Figure 1** and **Supplementary Table S1**.

Within *E. glareosa* s.l., the relationships among the populations were mostly poorly resolved, and in general, only the terminal clusters, including individuals from the same populations, had high BS (**Supplementary Figure S2**). The most divergent was population 165 from Armenia (BS 100%), followed by population 151 from Romania, although with BS <50%; these two populations also had deviating RGS. In addition, the single cluster with high BS (BS 99%) included populations 126–128 from Bulgaria that all had the lowest RGS. The K-means analysis (**Figure 5A**) showed the best separation into two groups: the western populations from the Pannonian Basin and the central Balkan Peninsula (Western Group) were separated from the eastern populations from the Thracian and Pontic steppes as well as from Turkey and Armenia (Eastern Group). At K = 3 and K = 4, only the Eastern group was further divided into subclusters, although without any clear pattern. This structure was also reflected in the NeighbourNet (**Figure 5B**).

### 3.4 Morphological differentiation

**Figure 6:**
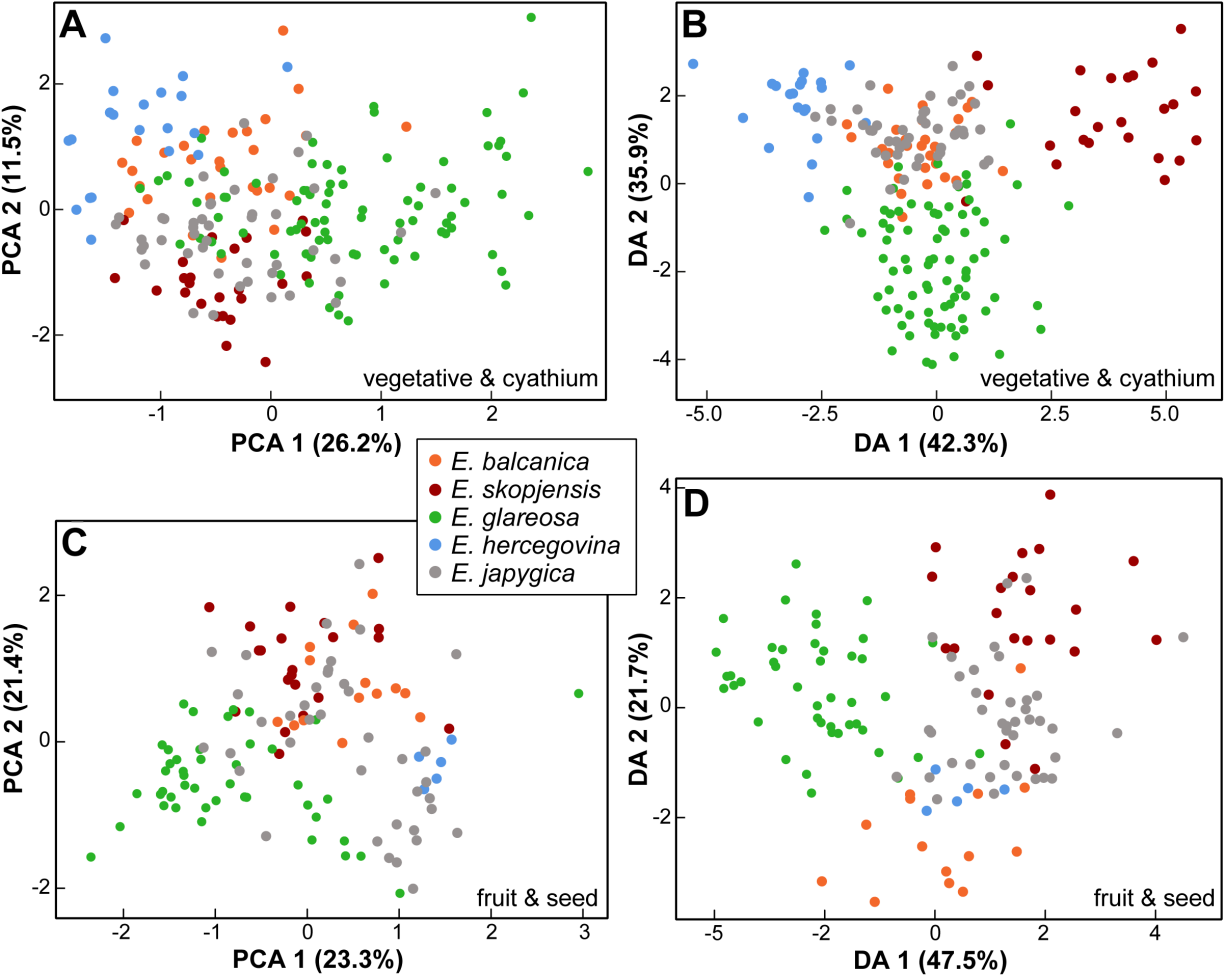
Morphological differentiation among *Euphorbia balcanica*, *E. skopjensis*, *E. glareosa* s.l., *E. hercegovina*, and *E. japygica*. **(A)** Principal component analysis (PCA) and **(B)** discriminant analyses (DA) based on vegetative and cyathium characters; **(C)** PCA and **(D)** DA based on fruit and seed characters.

For vegetative and cyathium characters, the principal component analysis (PCA) scatter plot, including all taxa (first three components explaining 26.2%, 11.5%, and 10.1% of the total variation), showed a weak separation trend of *E. hercegovina* and *E. skopjensis* along the first component, whereas *E. balcanica*, *E. glareosa* s.l., and *E. japygica* were intermediate, with considerable overlap among them (**Figure 6A**). The characters that contributed most to the separation along the first component were length of a ray leaf, length of a raylet leaf, distance from the base to the widest part of a raylet leaf, stem width, and number of branchings of the longest terminal ray. The discriminant analysis (DA; **Figure 6B**) showed a similar pattern, although with a clearer differentiation of *E. hercegovina* and *E. skopjensis* from the other taxa along the first discriminant axis (42.3% of the total variation). The characters that contributed most to this separation were stem length, stem width, length of a middle stem leaf, length of a raylet leaf, width of a raylet leaf, ratio distance from the base to the widest part of a raylet leaf/length of a raylet leaf, and length of cyathial involucrum. Along the second discriminant axis (35.9%), there was a separation trend of *E. glareosa* s.l. from *E. japygica* and *E. balcanica* that overlapped considerably. The characters that contributed most to this separation were length of a middle stem leaf, length of a raylet leaf, distance from the base to the widest part of a raylet leaf, ratio distance from the base to the widest part of a stem leaf/length of a stem leaf, ratio length/width of cyathial involucrum, and number of branchings of the longest terminal ray.

Classificatory discriminant analysis classified 180 out of 200 individuals (90%) to the correct group. Of 20 incorrectly classified individuals, one (out of 24) of *E. skopjensis* was classified as *E. glareosa* s.l., and one as *E. japygica*; three individuals (out of 28) of *E. balcanica* were classified as *E. glareosa* s.l., and three as *E. japygica*; four individuals (out of 83) of *E. glareosa* s.l. were classified as *E. japygica*, two as *E. balcanica*, and one as *E. skopjensis*; one individual (out of 20) of *E. hercegovina* was classified as *E. balcanica*; two (out of 45) individuals of *E. japygica* were classified as *E. balcanica* and two as *E. hercegovina*.

For fruit and seed characters, the PCA scatter plot including all taxa (first three components explaining 23.3%, 21.4%, and 12.5% of the total variation) showed a strong overlap among the taxa (**Figure 6C**). *Euphorbia balcanica* and *E. skopjensis* overlapped with *E. japygica*, whereas there was a separation trend of *E. glareosa* s.l. from *E. balcanica* and *E. skopjensis* along the second principal component. The characters that contributed most to the separation along this component were fruit width, caruncle length and width, and seed width. The DA (**Figure 6D**) showed a similar pattern, although with a clearer pattern of differentiation of *E. glareosa* s.l. from other taxa along the first discriminant axis (47.5%). The characters that contributed most to this separation were fruit length, fruit width, ratio of fruit length/fruit width, seed width, and caruncle length. Along the second discriminant axis (21.7%), there was a separation trend of *E. balcanica* from the other taxa, and of *E. hercegovina* from *E. skopjensis*. The characters that contributed most to this separation were distance from the base to the widest part of a seed, fruit width, fruit length, ratio distance from the base to the widest part of a seed/seed length, and seed width.

Classificatory discriminant analysis classified 116 out of 129 individuals (90%) to the correct group. Of 13 incorrectly classified individuals, one (out of 14) of *E. balcanica* was classified as *E. skopjensis*; two individuals (out of 21) of *E. skopjensis* were classified as *E. japygica* and one as *E. balcanica*; two individuals (out of 44) of *E. glareosa* s.l. were classified as *E. japygica*, one as *E. skopjensis*, and one as *E. hercegovina*; one individual (out of 5) of *E. hercegovina* was classified as *E. japygica*; two (out of 45) individuals of *E. japygica* were classified as *E. hercegovina*, one as *E. balcanica*, and one as *E. skopjensis*.

**Figure 7:**
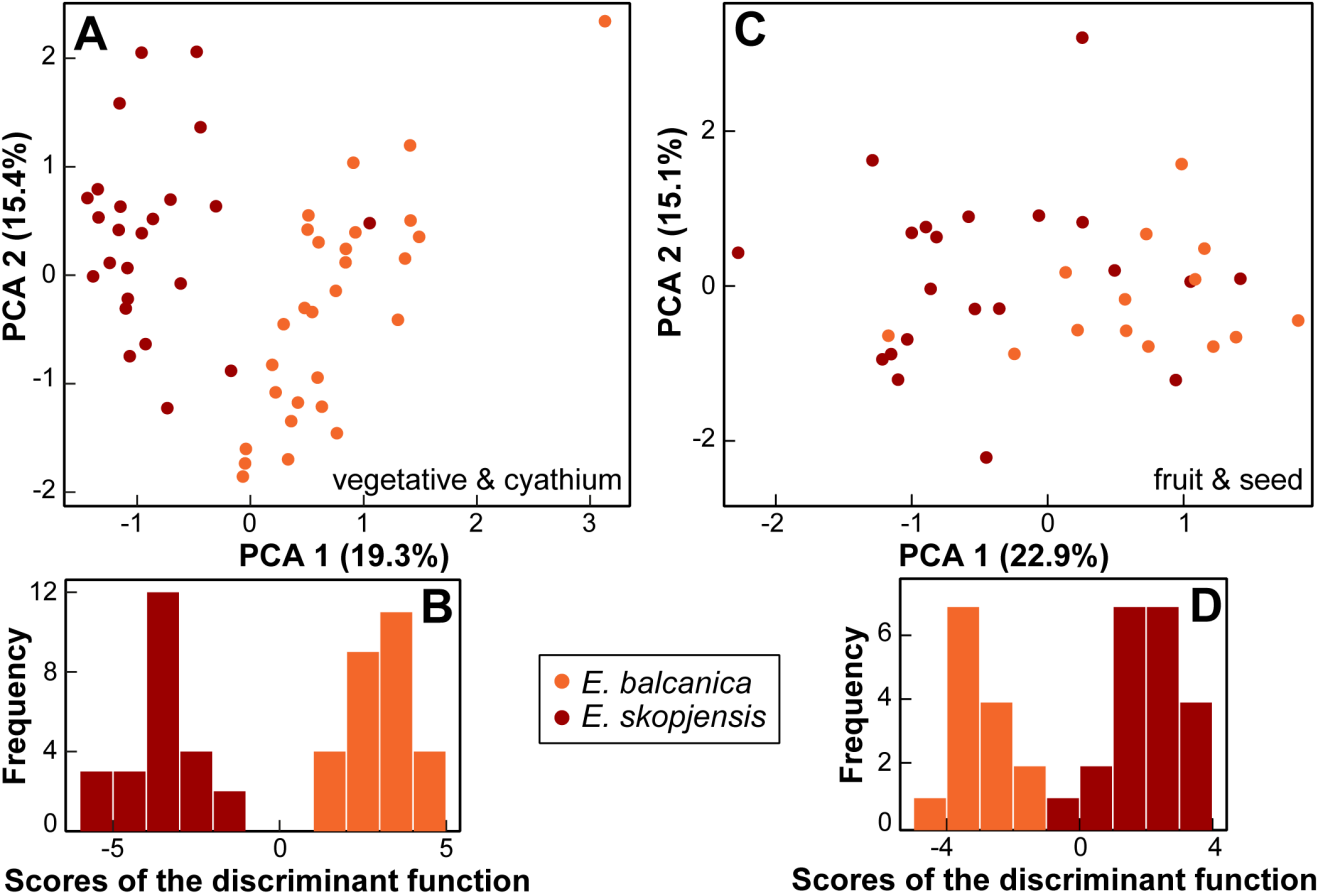
Morphological differentiation between *Euphorbia balcanica* and *E*. *skopjensis*. **(A)** Principal component analysis (PCA) and **(B)** discriminant analyses (DA) based on vegetative and cyathium characters; **(C)** PCA and **(D)** DA based on fruit and seed characters.

For vegetative and cyathium characters, the PCA scatter plot, including only *E. balcanica* and *E. skopjensis* (first three components explaining 19.3%, 15.4%, and 12.1% of the total variation), showed a clear separation between them along the first component (**Figure 7A**). An exception was one individual from population 102 of *E. skopjensis* that was positioned among samples of *E. balcanica*. The characters that contributed most to this separation were length of (the longest) terminal ray, length of a ray leaf, length of (the longest) fertile axillary ray, and distance from the base to the widest part of a ray leaf. In addition, the DA showed a clear differentiation between both species. The characters that contributed most to this separation were length of (the longest) terminal ray, length of a middle stem leaf, stem length, width of a raylet leaf, length of a raylet leaf, and the ratio length of cyathial involucre/width of cyathial involucre. Classificatory discriminant analysis classified all individuals to the correct group.

For fruit and seed characters, the PCA scatter plot, including only *E. balcanica* and *E. skopjensis* (first three components explaining 22.9%, 15.1%, and 14.0% of the total variation), showed a separation trend between them along the first component, although with considerable overlap (**Figure 7C**). The characters that contributed most to this separation were caruncle length, ratio of caruncle length/caruncle width, and ratio of seed length/seed width. In addition, the DA showed a clear differentiation between both species. The characters that contributed most to this separation were ratio of seed length/seed width, seed width, fruit width, and caruncle length. Classificatory discriminant analysis classified only a single individual of *E. skopjensis* incorrectly. Variation of selected characters that discriminate between *E. balcanica* and *E. skopjensis* as well as between them and closely related taxa is presented in boxplots (**Supplementary Figure S3**).

## 4 Discussion

### 4.1 Cryptic diversity in the central Balkan Peninsula and description of two new species

Our integrative taxonomic approach applying an array of complementary methods revealed cryptic diversity in the central part of the Balkan Peninsula rendering the description of two new species, *E. balcanica* and *E. skopjensis*, necessary. Divergence of a putatively polyploid population of *E. glareosa* s.l. from the Skopje Basin (North Macedonia) was previously revealed based on RADseq data (Stojilkovič et al., 2022). Our AFLP analyses (**Figure 4**) with extended geographic sampling confirmed that diploid populations from Albania, Kosovo, and North Macedonia, pertaining to *E. balcanica*, and tetraploid populations from Skopje Basin in North Macedonia, pertaining to *E. skopjensis*, form a lineage differentiated from all other constituents of the *E. nicaeensis* alliance.

*Euphorbia balcanica* and *E. skopjensis* appear most closely related to Anatolian *E. erythrodon*, *E. macroclada*, and *E. petrophila*, as well as to Pannonian–Pontic–Anatolian *E. glareosa* s.l., but the relationships among these lineages are poorly resolved in the AFLP tree. This was similar in the case of RADseq data, where different analyses resulted in partly different topologies (Stojilkovič et al., 2022). In the same line, *E. balcanica* and *E. skopjensis* are intermediate among three groups in the NeighbourNet based on ITS sequences (**Figure 3B**), namely, (1) *E. hercegovina*, *E. japygica*, and *E. nicaeensis*, (2) *E. erythrodon* and *E. macroclada*, as well as (3) *E. glareosa* s.l. The RGS data and chromosome counts (**Figure 2**) further revealed that *E. balcanica* is diploid, with an RGS of 0.90–0.95 intermediate between that of most samples of *E. glareosa* s.l., *E. erythrodon*, and *E. petrophila* that have an RGS of approximately 0.70, and the samples of *E. hercegovina*, *E. japygica*, and *E. macroclada* (as well as some of *E. glareosa* s.l.; see below) that have an RGS between 1.00 and 1.20 (**Figure 2A**). Further, given that the populations of *E. skopjensis* are positioned in the same clade with *E. balcanica* in the NJ tree based on AFLP (**Figure 4**) and that the RGS of *E. skopjensis* is double of that of *E. balcanica* (**Figure 2A**), *E. skopjensis* is likely an autotetraploid that originated from *E. balcanica*.

The intermediate RGS and the central phylogenetic position in the ITS NeighbourNet of *E. balcanica* as well as poorly resolved relationships in the AFLP NJ tree suggest that this species, and thus also its tetraploid derivate *E. skopjensis*, might be of a hybrid origin, likely between geographically proximate *E. hercegovina* or *E. japygica* on one side, and *E. glareosa* s.l. on the other. This is further supported by early divergence of *E. skopjensis* within the *E. nicaeensis* lineage in the maximum likelihood tree and its position between *E. hercegovina* and *E. japygica* in the species tree inferred with SNAPP, both based on RADseq data (Stojilkovič et al., 2022; results summarised in **Supplementary Figure S4**), which uncovered close relationships of *E. balcanica* and *E. skopjensis* to *E. hercegovina* and *E. japygica*. In addition, Bayesian clustering of RADseq data using fastSTRUCTURE indicated an admixed genetic pattern in *E. skopjensis*, with a share of its genome corresponding to the lineage including *E. hercegovina* and *E. japygica*, and a share derived from the lineage including *E. glareosa* s.l (Stojilkovič et al., 2022; **Supplementary Figure S4**). The combination of genetic and RGS data are thus in favour of our hypothesis that the *E. balcanica/E. skopjensis* lineage might be of hybrid origin between Mediterranean and steppe lineages of the *E. nicaeensis* alliance.

Our morphometric analyses showed that there is a strong overlap in morphology between *E. balcanica* and closely related species, especially *E. japygica*, whereas *E. skopjensis* is much better differentiated based on vegetative and cyathium characters (**Figure 6B**; **Supplementary Figure S3**). On the other hand, *E. skopjensis* overlaps strongly with *E. japygica* in fruit and seed characters, whereas *E. balcanica* is better differentiated (**Figure 6D**). It was shown earlier (Stojilkovič et al., 2022) that morphological differentiation only partly follows evolutionary trajectories in the *E. nicaeensis* alliance, which resulted in taxonomic lumping of morphologically similar but evolutionarily divergent entities of this alliance in the past (e.g., Radcliffe-Smith and Tutin, 1968; Kuzmanov, 1979; Greuter et al., 1986; Govaerts et al., 2000). The discordant patterns likely result from (1) adaptation to similar habitats within divergent phylogenetic lineages leading to similar morphology (e.g., between *E. nicaeensis* and *E. glareosa* s.l.) or (2) from adaptation to divergent ecological niches within the same evolutionary lineages resulting in divergent morphology of closely related species (e.g., between *E. hercegovina* and *E. japygica*). The latter is also the case in the two newly described species that are morphologically clearly divergent (**Figure 7**), likely as a result of adaptation to divergent environments.

*Euphorbia balcanica* grows at higher altitudes in nutrient-poor habitats, such as gravelly grasslands or scrublands over serpentines (in Albania and Kosovo) or dolomite (in North Macedonia), which resemble the habitats of *E. hercegovina* and *E. japygica* (Stojilkovič et al., 2022; Boschin et al., 2024). On the other hand, *E. skopjensis* thrives at lower elevations in subruderal habitats, such as abandoned meadows or pastures with deeper, nutrient-rich soils, which is closer to the ecology of *E. glareosa* s.l (Stojilkovič et al., 2022). This ecological and morphological divergence led to differential treatments of the populations of *E. balcanica* and *E. skopjensis* in the Flora of Macedonia (Micevski, 1998; Matevski et al., 2018). Whereas the former were treated as *E. hercegovina* (*sub E. barrelieri* Savi subsp. *hercegovina* (Beck) Kuzmanov), those of *E. skopjensis* were considered to belong to *E. nicaeensis* (incl. *E. glareosa* s.l.). On the other hand, the populations of *E. balcanica* from Albania were thought to belong to *E. nicaeensis* (incl. *E. glareosa* s.l.; Qosja et al., 1992; Barina, 2017).

### 4.2 Complex diversification of *Euphorbia glareosa* s.l.

Our extended sampling of *E. glareosa* s.l. compared to that of Stojilkovič et al. (2022) brings further evidence of complex diversification patterns within *E. glareosa* s.l. Complexity within this taxon was recognised already based on morphology, which led to recognition of different (infra)specific taxa (e.g., Prokhanov, 1949; Kuzmanov, 1979; Greuter et al., 1986; Geltman, 2009). In addition, Stojilkovič et al. (2022) have shown pronounced RGS variation within *E. glareosa* s.l. suggesting polyploidisations but also homoploid differentiation, as well as strong genetic differentiation among populations based on RADseq data.

Our RGS data, calibrated with a chromosome count of 2*n* = 2*x* = 18 (**Figure 2**), indicated that most sampled populations are diploid with RGS between 0.683 and 0.731 (black cross in **Figure 1**) and pertain to *E. glareosa* s.str. In addition, populations 116 from Serbia and 162 from Turkey that have RGS 1.401 and 1.436 (black cross with white margin in **Figure 1**), respectively, are likely autotetraploids derived independently from geographically close diploid populations; they should thus also be treated as *E. glareosa* s.str. as they are also morphological similar to the diploids. The independent origin of the two tetraploid populations is supported by RGS (**Figure 2A**) and AFLP (**Figure 5**) data. The RGS of the diploid populations 113 and 115 that are geographically closest to the putatively tetraploid population 116 (all south-east Serbia) is 0.667 and 0.699, respectively, which is half of the RGS of population 116. In addition, populations 115 and 116 are most closely related and share several common splits in the AFLP NeighbourNet (**Figure 5B**). On the other hand, the putatively tetraploid population 162 from Turkey is nested within the diploid populations 152 and 156 from the Pontic steppes in the AFLP NeighbourNet, which have RGS 0.726 and 0.718, respectively, i.e., half the RGS of population 162.

Phylogeographically, there is clear genetic divergence between the (1) Pannonian, central, and eastern Balkan populations, and (2) the easternmost Balkan, Pontic, and Anatolian populations of *E. glareosa* s.str. as indicated by the main AFLP split at K = 2 (**Figure 5A**). This split was evidenced also by the RADseq data, although based on a much scarcer geographic sampling (seven populations analysed; Stojilkovič et al., 2022) and corresponds to the border between zonal and extrazonal steppes (Wesche et al., 2016; Kirschner et al., 2020). Our study thus additionally highlights the outstanding conservation value of the extrazonal European steppes that are not just an outpost of zonal Eurasian steppes; many of their characteristic species evolved independently in isolation for most of their history (Kirschner et al., 2020).

Deviating RGS values (**Figure 2**; **Supplementary Table S1**) of populations 126–128 (RGS 0.592–0.601; white cross with black margin in **Figure 1**), were likely caused by other factors than polyploidisation. Alongside polyploidy, accumulation of retrotransposons and other repetitive elements is considered the main cause of GS increase in angiosperms (Pellicer et al., 2018). We observed also a partial morphological and ecological differentiation of these populations, which were growing on steeper, open gravelly, and stony grounds compared to *E. glareosa* s.str., which mostly grows on deeper soils. Our preliminary taxonomic assessment suggests that they likely belong to *E. dobrogensis*. These populations were also genetically clearly differentiated; they formed a well-supported (BS 99%) lineage in the AFLP NJ tree (**Supplementary Figure S2**) and the NeighbourNet (**Figure 5E**), and formed a separate cluster at K = 4 (**Figure 5C**).

On the other hand, the deviating RGS of populations 132, 151, and 165 (1.047–1.146), as well as of populations 133 and 154 (1.226, 1.257; grey cross with black margin in **Figure 1**), is likely connected both to polyploidisation and subsequent RGS differentiation. These populations were morphologically fairly variable and appeared similar to *E. dobrogensis* (populations 132, 151, and 154), *E. cadrilateri* (population 133), and *E. glareosa* s.str. (population 165). They all formed a separate AFLP cluster at K = 5 (**Figure 5D**), which was intermediate between the diploid populations 126–128 of *E.* cf. *dobrogensis* and the eastern populations of diploid *E. glareosa* s.str. in the AFLP NeighbourNet (**Figure 5E**). This, along with their deviating RGS and diverse morphology, suggests that they likely originated via polyploidisation, but their parental species might either have been diploid *E. dobrogensis* (i.e., autopolyploid origin), or *E. dobrogensis* and *E. glareosa* s.str. (i.e., allopolyploid origin). Alternatively, tetraploidisation of *E. dobrogensis* and subsequent hybridisation with tetraploid *E. glareosa* s.str. (not included in our limited sampling in the Pontic area) could have led to the same genetic and RGS patterns. All these populations were most early divergent within *E. glareosa* s.l. in the AFLP NJ tree (BS <50%; **Supplementary Figure S2**), which is also in line with the tree based on RADseq (Stojilkovič et al., 2022). There, only populations 151 and 165 were included and resolved as sister to all other populations pertaining to *E. glareosa* s.str. In summary, the intricate patterns within *E. glareosa* s.l. call for further studies, especially in the east of the distribution range, i.e., the Pontic area and Anatolia, where the group appears to be most diverse.

### 4.3 Taxonomic treatment

**Figure 8:**
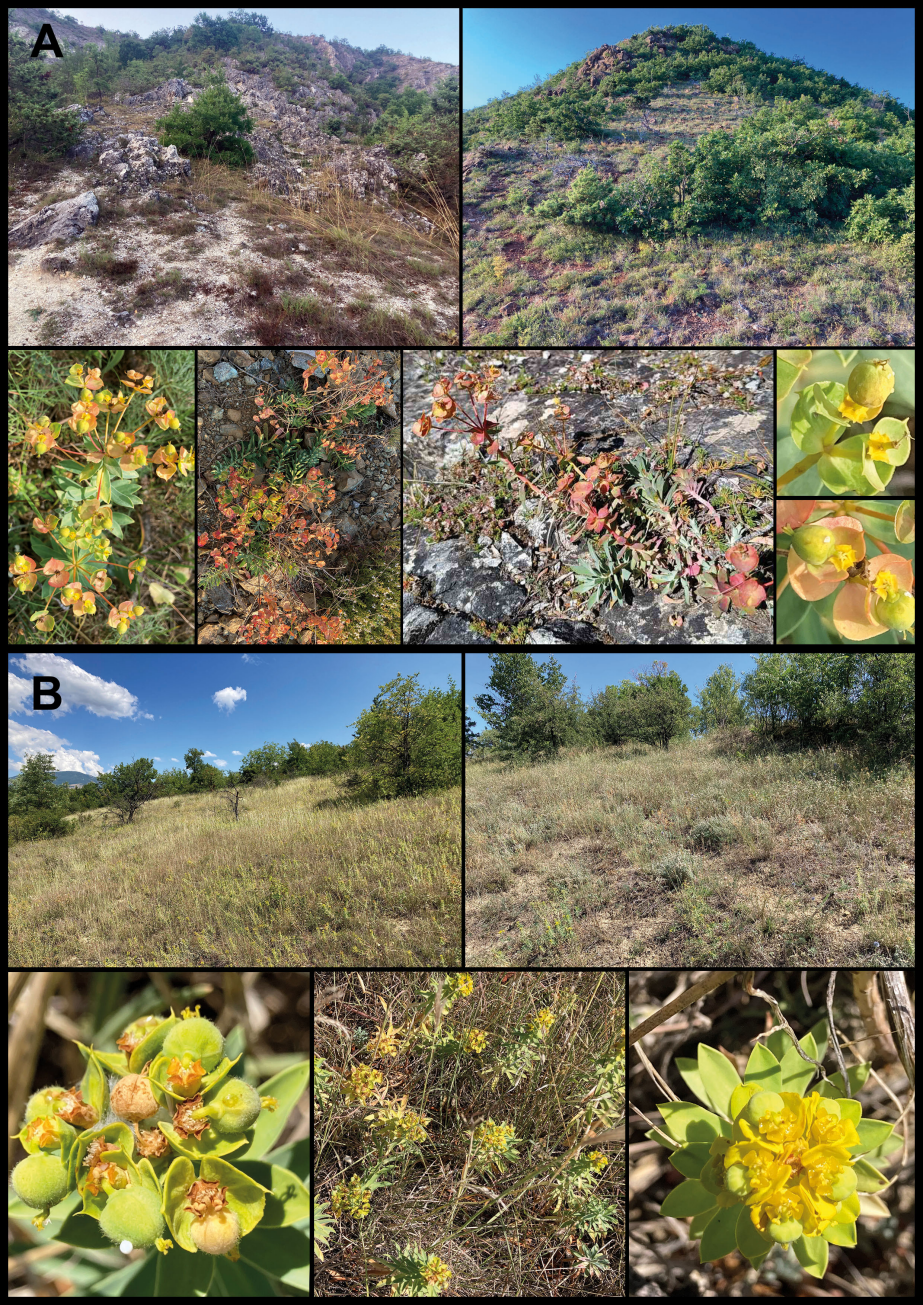
*Euphorbia balcanica* **(A)** and *E. skopjensis* **(B)** in their natural environments. Photos: Sharovikj Ivanova & Frajman.

Below, we provide a taxonomic treatment for the new species *E. balcanica* (**Figure 8A**) and *E. skopjensis* (**Figure 8B**), as well as a description of *E. glareosa* s.l. Despite the fact that some populations included in our study were preliminarily assigned to *E. dobrogensis* or *E. cadrilateri* (see Discussion above), we here refrain from taxonomic decisions about these two taxa; therefore, we do not list any heterotypic synonyms or infraspecific taxa of *E. glareosa* s.l. Further studies with extended geographic sampling in Anatolia and the Pontic areas are needed to clarify their taxonomic rank and status. Based on the available data, it appears reasonable to treat populations 126–128 with lower genome size as a distinct species, *E. dobrogensis*. In addition, the western AFLP cluster of *E. glareosa* s.str. pertains to *E. pannonica* and the eastern to *E. stepposa* [both to be treated as subspecies as suggested already by Geltman (2020)], but the relationships of the latter to *E. glareosa* s.str. (described from Crimea) as well as to the remaining taxa described from the Eurasian zonal steppes (*E. cadrilateri*, *E. goldei*, and *E. volgensis*) remain unresolved.

#### 4.3.1 Identification key to species of the central and eastern Balkan Peninsula

**1** Smaller decumbent plant, (8)11–29(38) cm high, with (1.0)1.5–3.0(4.0) mm-thick stems. Cauline leaves (1.2)1.4–2.9(3.6) × (0.3)0.4–0.7(1.1) cm, (2.2)2.7–4.6(5.1) times longer than wide. *Central Balkan Peninsula (but not Skopje Basin) on gravelly ground over serpentine or dolomite*……………………………………… ***E. balcanica***
1* More robust plant, mostly erect, (5)16–46(60) cm high, with (1.0)1.8–3.9(4.8) mm-thick stems. Cauline leaves (0.8)1.9–4.5(5.7) × (0.3)0.5–1.2(1.9) cm, (0.9)2.9–5.6(7.7) times longer than wide. *Mostly on deeper soils, not over serpentine, rarely over dolomite* …………………………**2**
**2** Terminal rays 3–6(9), the longest (0.5)1.0–2.7(3.6) cm long, once dichotomously branched. Fertile axillary rays (0)1–11(16). The longest fertile axillary ray (0.6)1.2–3.5(5.4)-cm long. Cauline leaves (1.5)2.4–5.0(5.7) × (0.4)0.5–1.0(1.4) cm, (3.2)3.8–6.3(7.4) times longer than wide. Ray leaves (0.6)0.7–1.2(1.9) × 0.5–1.2(1.3) cm, (0.8)0.9–1.8(2.7) times longer than wide. Nectarial glands sometimes with two horns. Seeds (2.1)2.2–2.8(3.0) × (1.2)1.6–2.0(2.2) mm. *Skopje Basin (North Macedonia)*…………… ***E. skopjensis***
2* Terminal rays (2)5–10(14), the longest (1.4)2.3–6.5(9.9) cm long, 1–3 times dichotomously branched. The longest fertile axillary ray (2.0)3.2–8.0(12.2) cm long. Cauline leaves (0.8)1.7–4.3(5.3) × (0.3)0.4–1.2(1.9) cm, (0.9)2.7–5.3(7.2) times longer than wide. Ray leaves (0.6)1.1–2.7(3.7) × (0.4)0.6–1.4(2.4) cm, (0.7)1.2–2.7(3.6) times longer than wide. Nectarial glands without horns. Seeds (1.6)1.9–2.5(3.4) × (1.1)1.3–1.7(1.8) mm. *Widespread in the Pannonian Plain, eastern Balkan Peninsula, Pontic and Anatolian steppes, Caucasus; rare in central Balkan Peninsula* ………………………………………………… ***E. glareosa***

***Euphorbia balcanica* Sharovikj Ivanova & Frajman, sp. nov**.

Type: “Flora of North Macedonia, Dolneni, Debreška Krasta northeast of the village Debrešte, 682 m, 21°19′51″E, 41°29′16″N; gravelly pasture and scrubland over dolomite. Leg. A. Sharovikj Ivanova 18525, 15.06.2024″ (holotype in W, isotypes in IB and MKNH).

*Description:* Glabrous and glaucous decumbent perennial, (8) 11–29(38) cm high, with (1.0)1.5–3.0(4.0) mm thick stems. Terminal rays (4)5–8(10), the longest (1.9)2.3–4.6(5.9) cm long, 1–2 times dichotomously branched. Fertile axillary rays 0–7(10), the longest (2.3)3.0–5.7(7.2) cm long. Cauline leaves pruinose–papillose, (1.2)1.4–2.9(3.6) × (0.3)0.4–0.7(1.1) cm, (2.2)2.7–4.6(5.1) times longer than wide, widest at (0.5)0.6–0.7(0.8) of their length, oblanceolate, with cuneate base and obtuse to mucronate apex; margin narrowly cartilaginous with dense, pointed papillae, minutely serrulate, especially toward the tips. Ray leaves broadly ovate to obovate, (0.7)0.9–1.5(2.5) × (0.5)0.6–1.3(1.7) cm, (0.8)1.0–2.0(2.3) times longer than wide, widest at (0.2)0.3–0.6 of their length. Raylet leaves broadly ovate–cordate, 0.6–1.2(1.5) × (0.7)0.9–1.5(1.7) cm, (0.5)0.6–0.9(1.0) times longer than wide, widest at (0.1)0.2–0.4(0.5) of their length, with shallowly cordate base and mucronate apex. Cyathial involucre campanulate, (1.9)2.3–3.1(4.0) × (1.2)1.6–2.5(3.0) mm, (0.9)1.0–1.6 times longer than wide. Cyathial lobes usually pubescent on the inner side. Nectarial glands broadly obovate–truncate to trapezoid or broadly semilunate, (0.5)0.7–1.8 × (0.9)1.2–1.7(2.0) mm, (0.4)0.5–1.4 times longer than wide, with 0–0.2(0.4) mm-deep emargination and mostly with two horns of different lengths. Fruits glabrous or pubescent, pruinose–papillose, broadly ovoid, (1.8)3.2–4.2(5.0) × (1.8)2.6–3.5(4.0) mm, (0.9)1.0–1.3 times longer that wide, styles (1.2)1.4–1.9 mm long. Seeds ovoid, smooth–papillose, brownish or greyish, (2.1)2.2–2.6(2.8) × (1.4)1.5–1.8(1.9) mm, (1.2)1.3–1.6 times longer than wide. Caruncle conical, (0.6)0.7–1.0 × (0.8)0.9–1.2(1.3) mm, (0.6)0.7–0.9(1.0) times longer than wide.

*Distribution:* Central-western Balkan Peninsula (Albania, Kosovo, and North Macedonia).

*Habitat:* Open rocky and gravelly, nutrient-poor grasslands and scrublands over serpentine (Albania, Kosovo) or dolomite (North Macedonia).

*Etymology:* We name this species after the Balkan Peninsula, where it is endemic in its central part.

***Euphorbia skopjensis* Sharovikj Ivanova & Frajman, sp. nov**.

Type: “Flora of North Macedonia, Skopje Basin, north of the village Kondovo, 320 m, 21°18′44″E, 42°0′54″N; ruderalised pasture and road margin. Leg. A. Sharovikj Ivanova 18532, 24.06.2024″ (holotype in W, isotypes in IB and MKNH).

*Description:* Glabrous and glaucous erect perennial, (16)18–46(51) cm high, with (1.9)2.3–3.2(4.5) mm thick stems. Terminal rays 3–6(9), the longest (0.5)1.0–2.7(3.6) cm long, once dichotomously branched. Fertile axillary rays (0)1–11(16), the longest (0.6)1.2–3.5(5.4) cm long. Cauline leaves pruinose–papillose, (1.5)2.4–5.0(5.7) × (0.4)0.5–1.0(1.4) cm, (3.2)3.8–6.3(7.4) times longer than wide, widest at (0.4)0.6–0.8 of their length, oblanceolate, with cuneate base and obtuse to mucronate apex; margin narrowly cartilaginous with dense, pointed papillae, minutely serrulate, especially toward the tips. Ray leaves elliptic to ovate, (0.6)0.7–1.2(1.9) × 0.5–1.2(1.3) cm, (0.8)0.9–1.8(2.7) times longer than wide, widest at (0.2)0.3–0.7 of their length. Raylet leaves broadly ovate–cordate, (0.4)0.5–0.8(1.1) × (0.4)0.6–1.1(1.4) cm, (0.5)0.6–0.9(1.0) times longer than wide, widest at (0.1)0.2–0.6(0.7) of their length, with shallowly cordate base and mucronate apex. Cyathial involucre campanulate, (1.3)2.6–3.6(3.9) × (1.5)1.7–3.4(4.1) mm, (0.6)0.9–1.7(1.9) times longer than wide. Cyathial lobes usually pubescent on the inner side. Nectarial glands broadly obovate–truncate to trapezoid, rarely broadly semilunate, (0.5)0.6–1.1(1.3) × (1.0)1.1–1.8(2) mm, (0.4)0.5–0.7(0.8) times longer than wide, with 0–0.2(0.4) mm-deep emargination, sometimes with two horns of different lengths. Fruits glabrous or pubescent, pruinose–papillose, broadly ovoid, (2.5)3.2–4.6(5.0) × (2.7)2.8–4.3(4.9) mm, (0.8)0.9–1.2(1.6) times longer that wide, styles (1.0)1.3– to 2.0(2.4) mm long. Seeds ovoid, smooth–papillose, brownish or greyish, (2.1)2.2–2.8(3.0) × (1.2)1.6–2.0(2.2) mm, (1.0)1.1–1.6(1.9) times longer than wide. Caruncle conical, (0.5)0.6–0.9(1.0) × (0.7)0.8–1.2(1.4) mm, (0.5)0.7–0.9(1.0) times longer than wide.

*Distribution:* Skopje Basin in North Macedonia.

*Habitat:* Abandoned meadows and pastures, scrublands, often with semi-ruderal character.

*Etymology:* We have named the species after the city of Skopje as it is only known from this city and its surroundings.

***Euphorbia glareosa*** Pall. ex M.Bieb. in Fl. Taur.-Caucas. 1: 373 (1808) ≡ *E. nicaeensis* subsp. *glareosa* (Pall. ex M.Bieb.) Radcl.-Sm. in Repert. Spec. Nov. Regni Veg. 79: 55 (1968) ≡ *Galarhoeus glareosus* (Pall. ex M.Bieb.) Prokh. in Trudy Kuibyshevsk. Bot. Sada 1: 40 (1941) ≡ *Tithymalus nicaeensis* subsp. *glareosus* (Pall. ex M.Bieb.) Soják in Čas. Nár. Mus., Odd. Přír. 152: 22 (1983).

*Note: Euphorbia glareosa* is here treated in the broader sense, including *E. cadrilateri* Prodan, *E. dobrogensis* Prodan, *E. pannonica* Host., and *E. stepposa* Zoz. The taxonomic status of these taxa as well as *E. goldei* Prokh. and *E. volgensis* Krysth. should be resolved in the future studies. For typification and possible synonyms, see also Geltman (2020).

*Description:* Glabrous, rarely pubescent, and glaucous erect, rarely decumbent perennial, (4)15–46(61) cm high, with (1.0)1.8–4.0(4.8) mm-thick stems. Terminal rays (2)5–10(14), the longest (1.4)2.3–6.5(9.9) cm long, 1–3 times dichotomously branched. Fertile axillary rays (0)1–11(16), the longest (2.0)3.2–8.0(12.2) cm long. Cauline leaves pruinose–papillose, (0.8)1.7–4.3(5.3) × (0.3)0.4–1.2(1.9) cm, (0.9)2.7–5.3(7.2) times longer than wide, widest at (0.3)0.5–0.7(0.8) of their length, elliptic to oblanceolate, with cuneate base and broadly acuminate to mucronate apex; margin narrowly cartilaginous with dense, rounded, or pointed papillae, minutely serrulate, especially toward the tips. Ray leaves broadly ovate to obovate, (0.6)1.1–2.7(3.7) × (0.4)0.6–1.4(2.4) cm, (0.7)1.2–2.7(3.6) times longer than wide, widest at (0.1)0.3–0.6(0.7) of their length. Raylet leaves broadly ovate–cordate, (0.6)0.7–1.4(1.9) × (0.8)0.9–1.6(2.5) cm, (0.5)0.7–1(1.4) times longer than wide, widest at (0.1)0.2–0.4(0.6) of their length, with shallowly cordate base and mucronate apex. Cyathial involucre campanulate, (1.8)2.3–4(4.9) × (0.9)1.5–2.3(2.7) mm, (1)1.3–2.3(3) times longer than wide. Cyathial lobes usually pubescent on the inner side. Nectarial glands broadly obovate–truncate to trapezoid or broadly semilunate, (0.5)0.6–1.0(1.6) × (0.6)1.0–1.6(2.2) mm, (0.4)0.5–0.8(0.9) times longer than wide, with 0–0.2(0.5) mm-deep emargination, without horns. Fruits glabrous or pubescent, pruinose–papillose, broadly ovoid, (2.4)2.8–4.5(5.5) × (2.1)2.6–3.3(3.5) mm, (0.8)0.9–1.6(1.9) times longer that wide, styles (0.8)1.2– to 2(2.2) mm long. Seeds ovoid, smooth–papillose, brownish or greyish, (1.6)1.9–2.5(3.4) × (1.1)1.3–1.7(1.8) mm, (1.2)1.3–1.6(2.0) times longer than wide. Caruncle conical, (0.3)0.4–0.7(1.0) × (0.4)0.7–1.0(1.2) mm, (0.4)0.5–0.8(1.0) times longer than wide.

*Distribution:* Pannonian Basin, central and eastern Balkan Peninsula, Pontic area north of the Black Sea, Crimea, Asia Minor, Armenian Highlands.

*Habitat:* Grasslands and scrublands.

## Data Availability

The datasets presented in this study can be found in online repositories. The names of the repository/repositories and accession number(s) can be found in the article/**Supplementary Material**.

## References

[B1] Arrigo N. Felber F. Parisod C. Buerki S. Alvarez N. David J.. (2010). Origin and expansion of the allotetraploid *Aegilops geniculata*, a wild relative of wheat. New Phytol. 187, 1170–1180. doi: 10.1111/j.1469-8137.2010.03328.x 20561204

[B2] Arrigo N. Tuszynski J. W. Ehrich D. Gerdes T. Alvarez N. (2009). Evaluating the impact of scoring parameters on the structure of intra-specific genetic variation using RawGeno, an R package for automating AFLP scoring. BMC Bioinf. 10, 1–14. doi: 10.1186/1471-2105-10-33 PMC2656475 19171029

[B3] Barina Z. (2017). Distribution atlas of vascular plants in Albania (Budapest: Hungarian Natural History Museum).

[B4] Boschin M. Schönswetter P. Frajman B. (2024). Genetic and morphological differentiation within *Euphorbia japygica* (Euphorbiaceae) suggests divergence of populations from the south-eastern Apennine Peninsula. Bot. J. Linn. Soc 205, 38–54. doi: 10.1093/botlinnean/boad066

[B5] Coupland R. T. (1993). “Ecosystems of the world 8B. Natural grasslands,” in Eastern hemisphere and résumé (Elsevier, Amsterdam).

[B6] Cresti L. Schönswetter P. Peruzzi L. Barfuss M. H. J. Frajman B. (2019). Pleistocene survival in three Mediterranean refugia: Origin and diversification of the Italian endemic *Euphorbia gasparrinii* from the *E. verrucosa* alliance (Euphorbiaceae). Bot. J. Linn. Soc 189, 262–280. doi: 10.1093/botlinnean/boy082

[B7] Đurović S. Schönswetter P. Niketić M. Tomović G. Frajman B. (2017). Disentangling relationships among the members of the *Silene saxifraga* alliance (Caryophyllaceae): Phylogenetic structure is geographically rather than taxonomically segregated. Taxon 66, 343–364. doi: 10.12705/662.4

[B8] ESRI (2021). ArcMAP: release 10. Environmental systems research institute. Redlands, CA: Environmental Systems Research Institute.

[B9] Evanno G. Regnaut S. Goudet J. (2005). Detecting the number of clusters of individuals using the software STRUCTURE: A simulation study. Mol. Ecol. 14, 2611–2620. doi: 10.1111/j.1365-294X.2005.02553.x 15969739

[B10] Frajman B. Pachschwöll C. Schönswetter P. (2014). Contributions to the knowledge of the flora of the Dinarides (Balkan Peninsula). Phyton 54, 27–46. doi: 10.12905/0380.phython54(1)2014-0027

[B11] Frajman B. Schönswetter P. (2011). Giants and dwarfs: Molecular phylogenies reveal multiple origins of annual spurges within *Euphorbia* subg. Esula. Mol. Phylogenet. Evol. 61, 413–424. doi: 10.1016/j.ympev.2011.06.011 21708275

[B12] Frajman B. Záveská E. Gamisch A. Moser T. The STEPPE Consortium Schönswetter P. (2019). Integrating phylogenomics, phylogenetics, morphometrics, relative genome size, and ecological niche modelling disentangles the diversification of Eurasian *Euphorbia segueriana* s.l. (Euphorbiaceae). Mol. Phylogenet. Evol. 134, 238–252. doi: 10.1016/j.ympev.2018.10.046 30415023

[B13] Geltman D. V. (2009). Spurges (*Euphorbia* L., Euphorbiaceae) of the boreal Eurasia. I. Section *Paralias* Dumort [In Russian with English summary. Novosti Sist. Vyssh. Rast. 41, 166–191.

[B14] Geltman D. V. (2020). A synopsis of *Euphorbia* (Euphorbiaceae) for the Caucasus. Novosti Sist. Vyssh. Rast 51, 43–78. doi: 10.31111/novitates/2020.51.43

[B15] Govaerts R. Frodin D. G. Radcliffe-Smith A. (2000). Checklist and bibliography of Euphorbiaceae 2 (Kew: Royal Botanic Gardens).

[B16] Greilhuber J. Ebert I. (1994). Genome size variation in *Pisum sativum* . Genome 37, 646–655. doi: 10.1139/g94-092 18470109

[B17] Greuter W. Burdet H. M. Long G. (1986). Med-Checklist 3. Palermo: Med-Checklist trust of OPTIMA.

[B18] Hamasha H. N. von Hagen B. Röser M. (2012). *Stipa* (Poaceae) and allies in the Old World: Molecular phylogenetics realigns genus circumscription and gives evidence on the origin of American and Australian lineages. Plant Syst. Evol. 298, 351–367. doi: 10.1007/s00606-011-0549-5

[B19] Hartigan J. A. Wong M. A. (1979). Algorithm AS 136: A k-means clustering algorithm. J. R. Stat. Soc C-App. 28, 100–108. doi: 10.2307/2346830

[B20] Huson D. H. Bryant D. (2006). Application of phylogenetic networks in evolutionary studies. Mol. Biol. Evol. 23, 254–267. doi: 10.1093/molbev/msj030 16221896

[B21] Kearse M. Moir R. Wilson A. Stones-Havas S. Cheung M. Sturrock S.. (2012). Geneious Basic: an integrated and extendable desktop software platform for the organization and analysis of sequence data. Bioinformatics 28, 1647–1649. doi: 10.1093/bioinformatics/bts199 22543367 PMC3371832

[B22] Kirschner P. Záveská E. Gamisch A. Hilpold A. Trucchi E. Paun O.. (2020). Long-term isolation of European steppe outposts boosts the biome’s conservation value. Nat. Commun. 11, 1–10. doi: 10.1038/s41467-020-15620-2 32327640 PMC7181837

[B23] Kuzmanov B. (1979). “Euphorbia”, in Flora reipublicae popularis bulgaricae 7. Ed. Kuzmanov B. (Academia scientiarum Bulgaricae, Sofia), 118–177.

[B24] Kuzmanović N. Comanescu P. Frajman B. Lazarević M. Paun O. Schönswetter P.. (2013). Genetic, cytological and morphological differentiation within the Balkan-Carpathian *Sesleria rigida* sensu *Fl. Eur.* (Poaceae): A taxonomically intricate tetraploid-octoploid complex. Taxon. 62, 458–472. doi: 10.12705/623.13

[B25] Lal R. (2004). Carbon sequestration in soils of Central Asia. Land Degrad. Dev. 15, 563–572. doi: 10.1002/ldr.624

[B26] Matevski V. Čarni A. Ćušterevska R. Kostadinovski M. Mucina L. (2018). Syntaxonomy and biogeography of dry grasslands on calcareous substrates in the central and southern Balkans. Appl. Veg. Sci. 21, 488–513. doi: 10.1111/avsc.12374

[B27] Micevski K. (1998). “Flora na Republika Makedonija (The flora of the Republic of Macedonia) 1-4,” in Skopje: Macedonian Academy of Sciences and Arts, 781–1113.

[B28] Nei M. Li W. H. (1979). Mathematical model for studying genetic variation in terms of restriction endonucleases. P. Natl. A. Sci. 76, 5269–5273. doi: 10.1073/pnas.76.10.5269 PMC413122 291943

[B29] Niketić M. Đurović S. Z. Tomović G. Schönswetter P. Frajman B. (2022). Diversification within ploidy-variable Balkan endemic *Cerastium decalvans* (Caryophyllaceae) reconstructed based on genetic, morphological and ecological evidence. Bot. J. Linn. Soc 199, 578–608. doi: 10.1093/botlinnean/boab037

[B30] Peart B. (2008). “Life in a working landscape: Towards a conservation strategy for the world’s temperate grasslands: Compendium of regional templates on the status of temperate grasslands,” in Temperate grasslands conservation initiative. Vancouver: IUCN/WCPA.

[B31] Pellicer J. Hidalgo O. Dodsworth S. Leitch I. J. (2018). Genome size diversity and its impact on the evolution of land plants. Genes 9, 88. doi: 10.3390/genes9020088 29443885 PMC5852584

[B32] Prodan I. (1936). Conspectul florei dobrogei. Partea a II-a. Bulet Acad. Înalte Stud. Agron. Cluj. 6, 202–259.

[B33] Prokhanov Y. I. (1949). “Genus 856. Euphorbia L.”, in Flora SSSR. Eds. Shishkin B. K. Bobrov E. G. (Akademii Nauk SSSR, Moskva-Leningrad), 233–378.

[B34] Qosja X. Paparisto K. Demiri M. Vangjeli J. (1992). Flora e shqipërisë 2 [The flora of Albania 2] (Tirana: Academy of Science of Albania).

[B35] Radcliffe-Smith A. (1982). “Euphorbia L.”, in Flora of Turkey, vol. 7 . Ed. Davis P. H. (Edinburgh University Press, Edinburgh), 571–630.

[B36] Radcliffe-Smith A. Tutin T. G. (1968). ““*Euphorbia* L.”,” in Flora europaea 2. Eds. Tutin T. G. Heywood V. H. Moore D. M. Valentine D. H. Walters S. M. Webb D. A. (Cambridge University Press, Cambridge), 213–226.

[B37] Rešetnik I. Záveská E. Grgurev M. Bogdanović S. Bartolić P. Frajman B. (2022). Stability in the south, turbulence toward the north: evolutionary history of *Aurinia saxatilis* (Brassicaceae) revealed by phylogenomic and climatic modelling data. Front. Plant Sci. 13. doi: 10.3389/fpls.2022.822331 PMC8964184 35360300

[B38] Ronquist F. Teslenko M. van der Mark P. Ayres D. L. Darling A. Höhna S.. (2012). MrBayes 3.2: Efficient bayesian phylogenetic inference and model choice across a large model space. Syst. Biol. 61, 539–542. doi: 10.1093/sysbio/sys029 22357727 PMC3329765

[B39] RStudio Team (2022). RStudio: Integrated development for R (Boston, MA: RStudio PBC). Available at: http://www.rstudio.com/ (Accessed May 12 2024).

[B40] Schönswetter P. Suda J. Popp M. Weiss-Schneeweiss H. Brochmann C. (2007). Circumpolar phylogeography of *Juncus biglumis* (Juncaceae) inferred from AFLP fingerprints, cpDNA sequences, nuclear DNA content and chromosome numbers. Mol. Phylogenet. Evol. 42, 92–103. doi: 10.1016/j.ympev.2006.06.016 16905337

[B41] Skokanová K. Hodálová I. Mereďa P. Slovák M. Kučera J. (2019). The *Cyanus tuberosus* group (Asteraceae) in the Balkans: biological entities require correct names. Plant Syst. Evol. 305, 569–596. doi: 10.1007/s00606-019-01576-4

[B42] Španiel S. Rešetnik I. (2022). Plant phylogeography of the Balkan Peninsula: Spatiotemporal patterns and processes. Plant Syst. Evol. 308, 38. doi: 10.1007/s00606-021-01756-3

[B43] Stojilkovič V. Záveská E. Frajman B. (2022). From western Asia to the Mediterranean Basin: diversification of the widespread *Euphorbia nicaeensis* alliance (Euphorbiaceae). Front. Plant Sci. 13. doi: 10.3389/fpls.2022.815379 PMC9262032 35812903

[B44] Suda J. Trávníček P. (2006). Estimation of relative nuclear DNA content in dehydrated plant tissues by flow cytometry. Curr. Protoc. Cytom. 38, 7.30.1–7.30.14. doi: 10.1002/0471142956.cy0730s38 18770844

[B45] Swofford D. L. (2002). Phylogenetic analysis using parsimony (Sunderland, MA: Sinauer Associate). doi: 10.1111/j.0014-3820.2002.tb00191.x

[B46] Tomović G. Niketić M. Lakušić D. Ranđelović V. Stevanović V. (2014). Balkan endemic plants in Central Serbia and Kosovo regions: Distribution patterns, ecological characteristics, and centres of diversity. Bot. J. Linn. Soc 176, 173–202. doi: 10.1111/boj.12197

[B47] Van de Peer Y. de Wachter R. (1997). Construction of evolutionary distance trees with TREECON for Windows: Accounting for variation in nucleotide substitution rate among sites. Bioinformatics. 13, 227–230. doi: 10.1093/bioinformatics/13.3.227 9183524

[B48] Vos P. Hogers R. Bleeker M. Reijans M. Van de Lee T. Hornes M.. (1995). AFLP: a new technique for DNA fingerprinting. Nucleic Acids Res. 23, 4407–4414. doi: 10.1093/nar/23.21.4407 7501463 PMC307397

[B49] Wesche K. Ambarlı D. Kamp J. Török P. Treiber J. Dengler J. (2016). The Palearctic steppe biome: a new synthesis. Biodivers. Conserv. 25, 2197–2231. doi: 10.1007/s10531-016-1214-7

[B50] Záveská E. Maylandt C. Paun O. Bertel C. Frajman B. Schönswetter P.. (2019). Multiple auto-and allopolyploidisations marked the Pleistocene history of the widespread Eurasian steppe plant *Astragalus onobrychis* (Fabaceae). Mol. Phylogenet. Evol. 139, 106572. doi: 10.1016/j.ympev.2019.106572 31351183

