# Supplementary material for: Integrative taxonomy reveals cryptic diversity within the Euphorbia nicaeensis alliance (Euphorbiaceae) in the central Balkan Peninsula
Source: Front Plant Sci. 2025 Apr 14;16:1558466. doi: 10.3389/fpls.2025.1558466 (PMC12035539; doi:10.3389/fpls.2025.1558466)
Supplement: Supplementary file 1 [file DataSheet1.pdf]

Supplementary Material

Integrative taxonomy reveals cryptic diversity within the *Euphorbia nicaeensis* alliance (Euphorbiaceae) in the central Balkan Peninsula

Angela Sharovikj Ivanova[1,2], Peter Schönswetter[2], Mitko Kostadinovski[1], Michael H. J. Barfuss[3*], Renata Ćušterevska[1], Božo Frajman[2*]

[1]*Institute of Biology, Faculty of Natural Sciences and Mathematics, Ss. Cyril and Methodius University Skopje, Arhimedova 3, 1000 Skopje, North Macedonia*

[2]*Department of Botany, University of Innsbruck, Sternwartestraße 15, 6020 Innsbruck, Austria*

[3]*Department of Botany and Biodiversity Research, University of Vienna, Rennweg 14, 1030 Vienna, Austria*

[*]Corresponding authors: bozo.frajman@uibk.ac.at, michael.h.j.barfuss@univie.ac.at

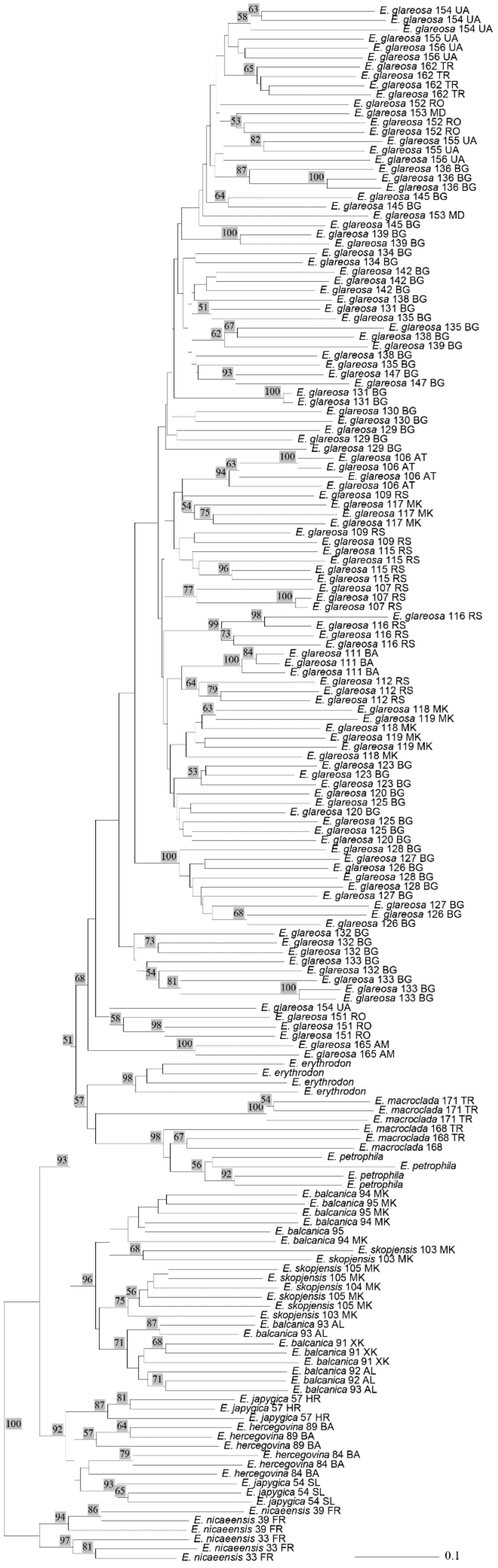

Supplementary Figure S1. Neighbour Joining tree based on the complete AFLP data showing the phylogenetic position of *Euphorbia balcanica* and *E. skopjensis* within the *E. nicaeensis* alliance. Numbers above branches are bootstrap values > 50. Population numbers correspond to Figure 1 and Supplementary Table S1.

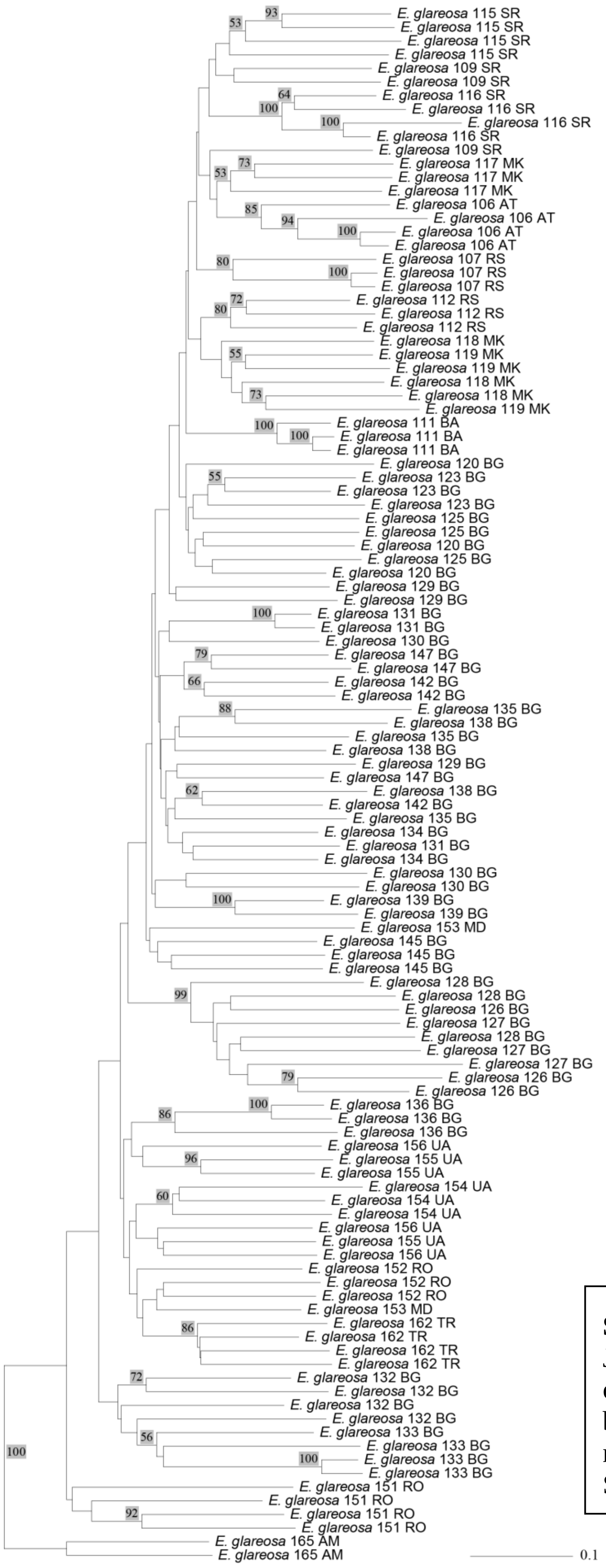

Supplementary Figure S2. Neighbour Joining tree based on the AFLP data including only *Euphorbia glareosa* s.l. Numbers above branches are bootstrap values > 50. Population numbers correspond to Figure 1 and Supplementary Table S1.

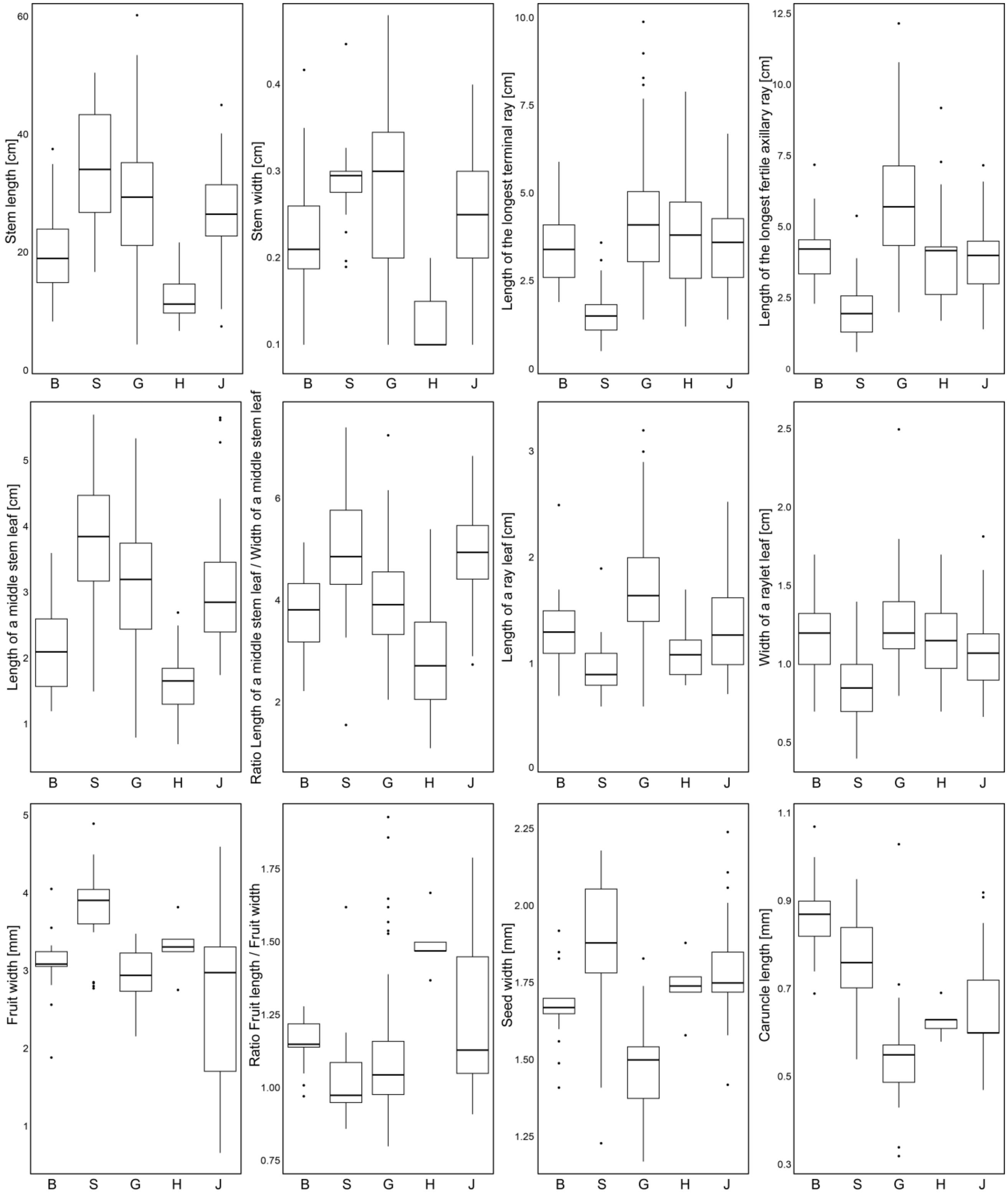

Supplementary Figure S3. Variation in selected morphological characters that differentiate between *Euphorbia balcanica* (B) and *E. skopjensis* (S), and/or between them and closely related *E. glareosa* (G), *E. hercegovina* (H) and *E. japygica* (J).

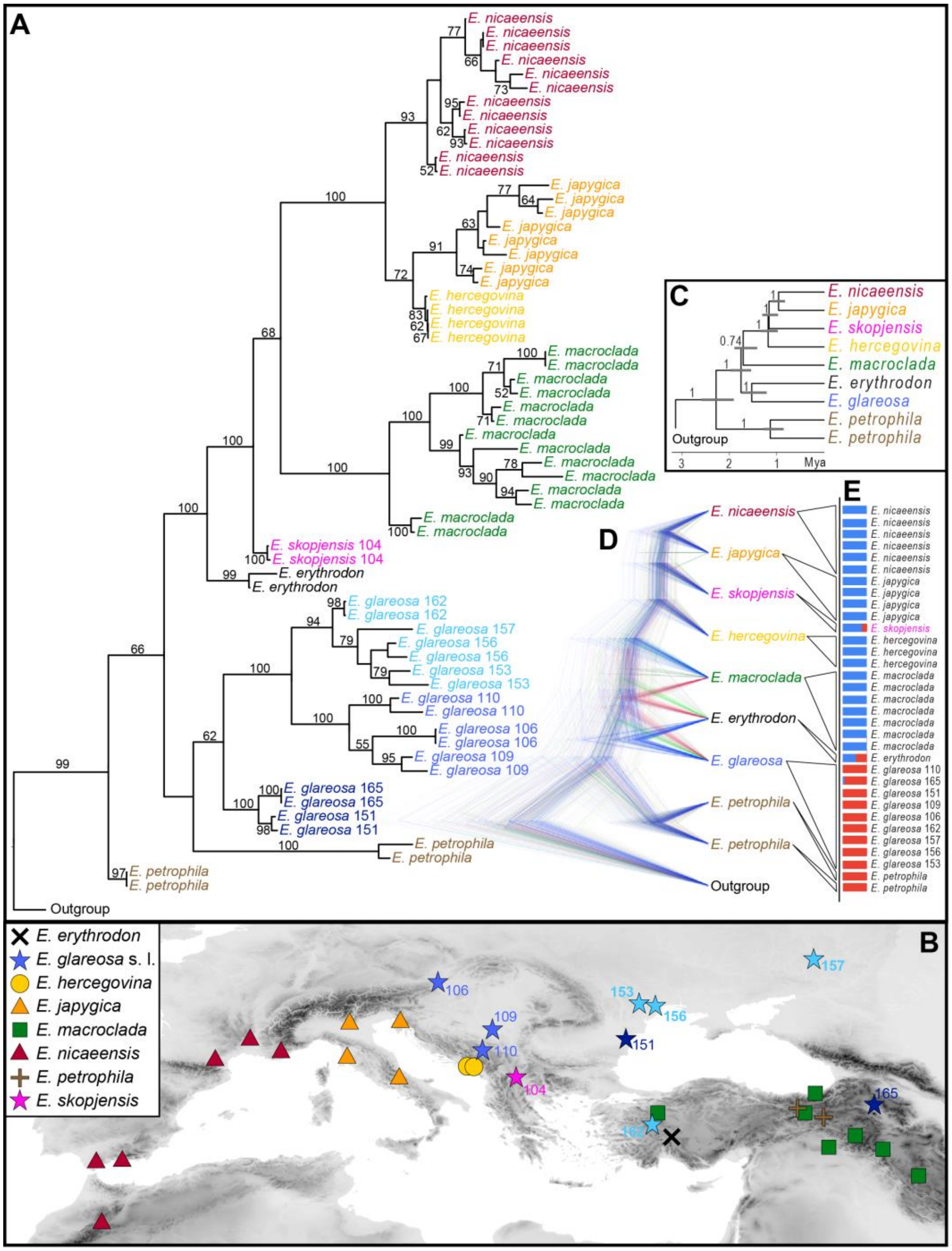

Supplementary Figure S4. Phylogenetic relationships within the *Euphorbia nicaeensis* alliance as inferred by RAD sequencing data in the study of Stojilkovič et al. (2022), showing the position of *E. skopjensis* (population 104). The figures were modified, the names adapted to current taxonomy, and population numbers to this study (Figure 1 and Supplementary Table S1). (A) maximum likelihood tree based on complete RADseq dataset; bootstrap values above 50% are indicated. (B) Geographic provenance of the investigated populations with colour coding as in A. (C) Time-calibrated species tree inferred with SNAPP and based on reduced RADseq dataset. Numbers above branches are posterior probabilities and the horizontal bars correspond to 95% highest posterior densities (HPD) of the age estimates. (D) Alternative topologies visualized with DensiTree and represented by different colours. (E) Division of all populations into two groups (blue and red) with Bayesian clustering using fastSTRUCTURE.
